# Endocannabinoid dynamics across marathon and ultramarathon running: evidence from two field studies

**DOI:** 10.1186/s12916-026-05186-z

**Published:** 2026-09-01

**Authors:** Michael Siebers, Dana Huvermann, Christoph Siebers, Deborah Canales-Romero, Alexandra Florea-Ghile, Lina Keite, Lucas John, Moritz Munk, Roman Bizjak, Jens Witzel, Sebastian Schulz, Johannes Kirsten, Laura Bindila, Anke Hinney, Marijke Grau, Daniel A Bizjak, Harald Engler, Johannes Fuss

**Affiliations:** 1 Institute for Forensic Psychiatry and Sexual Research, Center for Translational Neuro- and Behavioral Sciences, University of Duisburg-Essen, Essen, Germany; 2 Department of Psychiatry and Psychotherapy, Center for Translational Neuro- and Behavioral Sciences, LVR University Hospital Essen, University of Duisburg- Essen, Essen, Germany; 3 Department of Internal Medicine, Division of Sports and Rehabilitation Medicine, University Hospital Ulm, Ulm, Germany; 4 Department of Central IT, Division Applications and Databases, University of Zurich, Zurich, Switzerland; 5 Clinical Lipidomics Unit, Institute of Physiological Chemistry, University Medical Center of the Johannes Gutenberg University Mainz, Mainz, Germany; 6 Section of Molecular Genetics in Mental Disorders, Center for Translational Neuro- and Behavioral Sciences, Institute of Sex and Gender-Sensitive Medicine, University of Duisburg-Essen, Essen, Germany; 7 Institute of Cardiovascular Research and Sports Medicine, Molecular and Cellular Sports Medicine, German Sport University Cologne, Cologne, Germany; 8 Institute of Medical Psychology and Behavioral Immunobiology, Center for Translational Neuro- and Behavioral Sciences, University Hospital Essen, University of Duisburg-Essen, Essen, Germany

**Keywords:** Runner’s high, Endurance exercise, Endocannabinoid system, Neuromodulation, Physical activity, Mood regulation

## Abstract

**Background:**

Endocannabinoid (eCB) signaling has been implicated in the physiological and affective responses to endurance exercise, including phenomena such as the runner’s high. However, although humans have the capacity to run for several hours and even days, evidence regarding eCB signaling is largely limited to exercise bouts shorter than 60 min. Consequently, the temporal dynamics of eCB signaling during prolonged running and the accompanying acute affective responses remain unclear.

**Methods:**

This study investigated eCB signaling during long-distance running and following a 45-minute break. Two studies were conducted: In Study 1, 19 trained runners completed both a marathon and a duration-matched walking session, with repeated blood sampling every 14 km during the marathon and after a 45-minute recovery. In Study 2, 36 ultramarathon runners completed races of 100 km, 160 km, or 230 km and provided blood samples before and after their respective races. Plasma concentrations of anandamide (AEA), 2-arachidonoylglycerol (2-AG), 1-AG, arachidonic acid (AA), and palmitoylethanolamide (PEA) were quantified by a standardized liquid chromatography/multiple reaction monitoring assay. Euphoria, anxiety, and pain were assessed as core features of the runner’s high using visual analog scales.

**Results:**

AEA increased progressively throughout the marathon and remained elevated after 45 min, whereas walking elicited only modest changes. In line, after all ultramarathon distances AEA levels were increased compared with baseline. By contrast, an increase in 2-AG during exercise was observed only in the regular marathon, where concentrations rose significantly during the later stages of running and into early recovery. Elevated post-race 2-AG levels were also observed following all ultramarathon distances, consistent with a delayed, recovery-related response. Marathon running was associated with higher euphoria and lower anxiety than walking, while pain increased after 28 km of running. Ultramarathon running increased pain, reduced anxiety, and did not significantly alter euphoria post-exercise.

**Conclusions:**

Together, these findings show robust, time-dependent changes in circulating eCB concentrations during and after prolonged endurance running, as well as gradual increases in AEA during walking. These eCB dynamics occurred alongside acute affective changes during sustained endurance exercise.

**Supplementary Information:**

The online version contains supplementary material available at https://doi.org/10.1186/s12916-026-05186-z.

## Background

Humans are among the few mammals capable of sustained long-distance running, comparable to species such as dogs and horses [1]. Experimental studies have shown that voluntary running behavior in mice is influenced by intact endocannabinoid (eCB) signaling pathways [2, 3]. Whereas experimental animal studies have enabled the investigation of eCB dynamics over extended periods of voluntary running, human studies have largely been limited to short-duration exercise bouts lasting less than 60 min [4]. Consequently, little is known about the behavior of the eCB system during prolonged endurance exercise under real-world conditions.

This gap is particularly relevant in the context of the runner’s high, a phenomenon reported by some individuals during endurance exercise and characterized by euphoria, reduced anxiety, diminished pain, and post-exercise sedation [5]. Some individuals report experiencing time distortion and a state of flow while engaging in endurance activities. In the 1990s, the discovery of the eCB system led to an alternative hypothesis explaining the runner’s high [5, 6]. The eCB system includes anandamide (N-arachidonoylethanolamine, AEA), 2-arachidonoylglycerol (2-AG), the less active positional isomer 1-arachidonoylglycerol (1-AG), and related lipids such as N-palmitoylethanolamide (PEA) [6, 7]. While AEA and 2-AG primarily act via cannabinoid receptors CB1 and CB2, PEA exerts its effects mainly through non-cannabinoid targets, including peroxisome proliferator-activated receptor-α (PPAR-α) [8]. eCBs are synthesized on demand from membrane phospholipid precursors, primarily via N-acyl phosphatidylethanolamine-specific phospholipase D (NAPE-PLD) for AEA and diacylglycerol lipase (DAGL) for 2-AG. They are degraded by specific enzymes: fatty acid amide hydrolase (FAAH) hydrolyzes AEA to arachidonic acid (AA), whereas monoacylglycerol lipase (MAGL) converts 2-AG into AA. Unlike endorphins, eCBs are lipophilic and can cross the blood-brain barrier [5]. Therefore, peripherally measured eCBs reflect neuromodulatory processes occurring in the brain.

The runner’s high is most commonly associated with moderate-intensity endurance exercise below the anaerobic threshold [9]. The most pronounced positive mood effects have previously been observed after approximately 35 min of endurance exercise [10]. The concept of the runner’s high was first described in the 1970s. Early research attributed the runner’s high to the release of endorphins, and the theory was widely supported [11, 12]. However, endorphins bind to opioid receptors and are large, hydrophilic molecules. There is currently no convincing evidence that they cross the blood–brain barrier to a relevant extent. Furthermore, studies based on peripheral blood sampling have yielded inconsistent results and have provided only limited insights into the neurochemical basis of this phenomenon [5, 10, 13]. In contrast, several human brain imaging studies have indicated opioid system activation during exercise; however, its role appears to be most prominent during prolonged (~ 2 h) and high-intensity endurance exercise [14–18].

Subsequent research found that eCB levels increase significantly during running and, to a lesser extent, during cycling [19, 20]. While their exact origin during endurance running remains incompletely understood, eCBs are generally considered to be synthesized “on demand” from membrane phospholipid precursors in multiple tissues, including the vasculature, skeletal muscle, and the brain [2, 21, 22]. However, the specific sources and regulatory mechanisms underlying exercise-induced changes in circulating eCB levels remain unclear, and peripheral measures should therefore be interpreted as an indirect reflection of these processes.

Our previous research focused on the question of whether eCBs or endorphins mediate the runner’s high. In a mouse study, we were able to show that two key features of the runner’s high (anxiolysis and hypoalgesia) depend on the eCB system rather than the opioid system by blocking both pathways pharmacologically and through genetic mutagenesis [2]. In the next step, we aimed to reproduce our results in humans. We conducted a follow-up randomized, double-blind, placebo-controlled trial involving 63 participants running and walking on a treadmill for 45 min [23]. Half of the participants received 50 mg of naltrexone, a central opioid antagonist, while the other half received a placebo. Both groups reported similar self-assessed runner’s high scores, indicating that the runner’s high occurred independently of opioid levels. In contrast, running induced significantly elevated eCB levels, which were accompanied by increased runner’s high ratings. Walking for 45 min, however, led to smaller increases in eCB levels and did not produce comparable euphoric or anxiolytic effects, suggesting a potential role of eCBs in mediating the runner’s high. In a systematic review, we found that eCB release after endurance sports is a robust finding [10]. Long-term sports programs have been shown to reduce baseline eCB levels, suggesting a potential adaptive mechanism [10].

In the present study, we aimed to close the research gap regarding the dynamics of eCBs in endurance exercise lasting longer than 60 min. To date, only one study has examined the relationship between changes in energy metabolism and endocrine alterations: in 18 ultramarathon runners participating in a 100-km race, with incomplete race distances due to non-finishers, the authors observed increases in AEA and related lipids, but not in 2-AG [24]. However, the study did not provide clarity on the temporal dynamics of eCB signaling and included both finishers and non-finishers. To examine these processes more closely under real-world endurance running conditions, we conducted a marathon study (Study 1) and an ultramarathon study (Study 2).

Our aims were threefold: first, to examine how eCB signaling evolves during long-distance running in real-world running conditions; second, to assess the persistence of eCB levels after a post-marathon 45-minute recovery period; and third, to evaluate whether core psychological features of the runner’s high, namely euphoria, anxiety, and pain, which have been associated with eCB release, would also be affected by prolonged endurance exercise. We conducted a marathon field study using a within-subject design to compare prolonged running and walking in nature (Study 1). Based on prior findings, we expected eCB levels to continuously increase throughout running and to decline after a 45-minute recovery period [10]. To explore whether the findings from the marathon study would also be relevant in more extreme endurance conditions, we studied participants in the TorTour de Ruhr^®^ (TTdR) ultramarathon, in which 36 athletes completed distances of 100 km, 160 km, or 230 km (Study 2). We hypothesized that eCB levels would be elevated compared with baseline.

## Methods

### Study 1: marathon study

#### Participants

We conducted a marathon-walking study at Lake Baldeney in Essen, Germany. A total of 21 individuals were recruited for the study through internet forums, word of mouth, and announcements in running shops. Following telephone screening, 20 participants were considered eligible based on inclusion and exclusion criteria. One individual was excluded due to insufficient running experience (inability to complete a marathon distance). Of the 20 participants initially enrolled, one participant was excluded from the final analysis due to non-attendance at the second (walking) session, resulting in a final sample of *n* = 19. Inclusion criteria included the ability to run a marathon, fluency in German, and being experienced runner aged 18–60 years not taking any prescription medication (except contraceptives). Running experience was defined as the ability to complete a marathon distance, which was assessed during a structured pre-study telephone screening based on participants’ reported training routines and previous marathon completion. Exclusion criteria involved pre-existing medical conditions, drug use including cannabis use within four weeks prior to testing, pregnancy, breastfeeding, and a history of severe psychiatric or somatic disease. These criteria were verified through a pre-study telephone screening, during which participants also received detailed study information and an informed consent brochure via email.

#### Experimental procedure

Participants were instructed to abstain from caffeine and nicotine for the entire study day prior to testing and to avoid eating for at least two hours before testing. They were also asked not to run for at least 24 h prior to both sessions. Testing sessions were scheduled on weekends at noon to minimize diurnal fluctuations in cortisol and eCB levels [25]. Upon arrival, exclusion criteria were reviewed again. Participants were briefed about the study, and written informed consent was obtained.

Volunteers completed standardized questionnaires, including the International Physical Activity Questionnaire (IPAQ) [26], Exercise Dependence Scale (EDS-R) [27], and a custom sociodemographic survey. This survey gathered data on age, sex, health conditions, medication and drug use, exercise habits, and socioeconomic status.

Physical assessments included measurements of body mass index (BMI) and body fat percentage using an Omron BF 511 (Omron Healthcare, a division of the Omron Corporation, Kyoto, Japan). Emotional states were evaluated using visual analog scales (VAS) assessing anger, euphoria, happiness, anxiety, sedation, pain, energy, tension, and dejection, rated from 0 (none) to 100 (high) as used in other studies [14, 23]. Participants were equipped with GPS running watches (Polar Pacer Pro, Polar Electro Oy, Kempele, Finland), and baseline blood samples were collected. Heart rate (HR) data during the blood sampling were defined as periods with minimal movement (< 2 km/h) around the recorded time of the blood sampling and removed together with data points in the preceding and following 30 s.

The marathon session involved three 14 km loops around Lake Baldeney in Essen completed at participants’ preferred pace. Blood samples were collected and runner’s high ratings were assessed at the beginning and after each loop using VAS ratings. Following the marathon, participants took a 45-minute break for showers and refreshments. After the break, a final blood sample was collected, and runner’s high ratings were reassessed. Participants were also asked if they had experienced a runner’s high (yes/no/uncertain) during the marathon. No formal definition of the runner’s high was provided, and responses were therefore based on participants’ individual understanding of the concept. A concluding medical interview assessed participants’ health before discharge from the study site, including the presence of any adverse events, physical complaints, or signs of excessive fatigue.

**Fig. 1:**
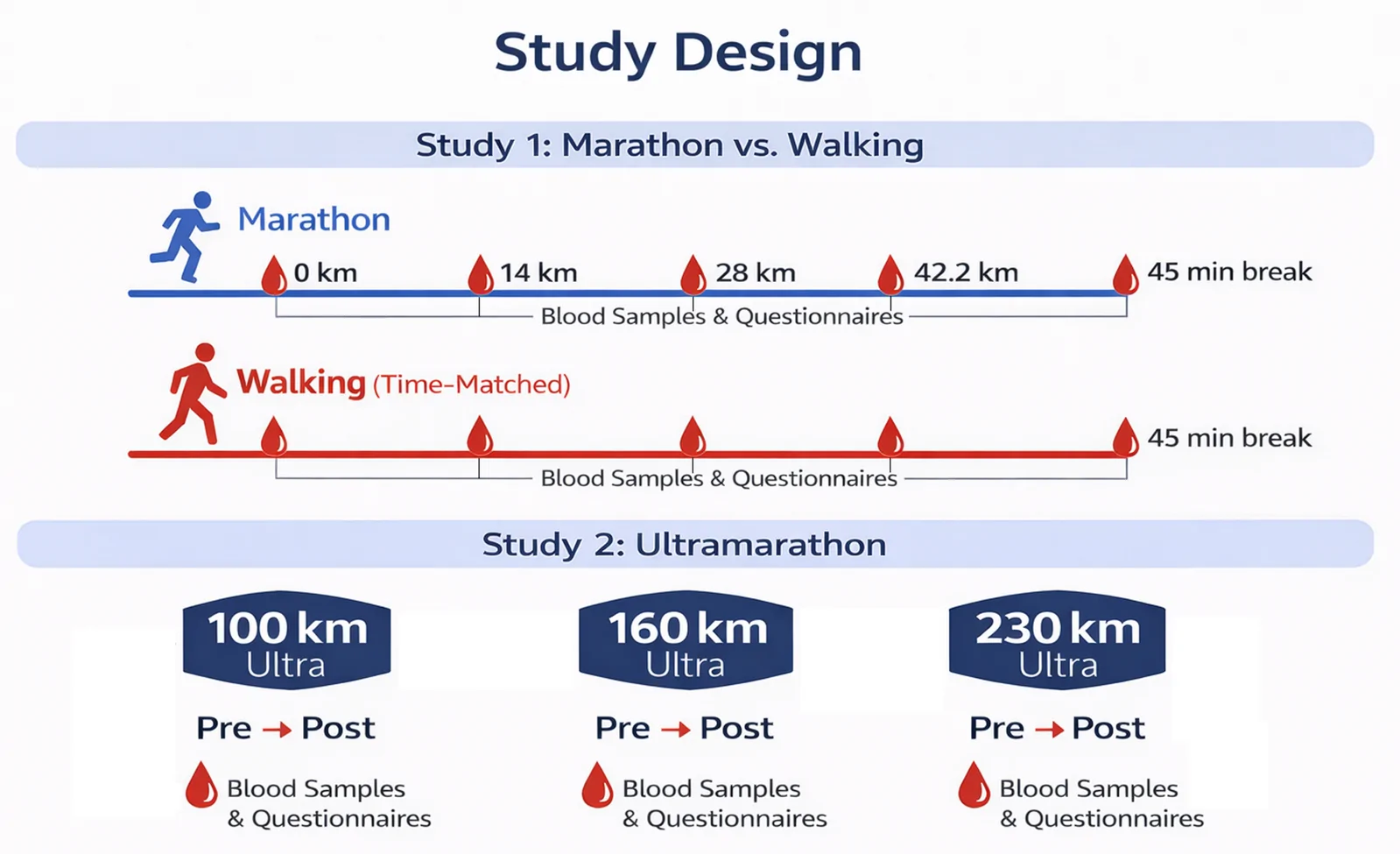
Study design and timing of assessments in the marathon and ultramarathon investigations. Study 1 (Marathon vs. Walking): Participants completed a marathon running condition (blue) and a time-matched walking condition (red). Blood samples and questionnaires were collected at baseline (0 km), after 14 km, 28 km, and at the end of the marathon (≈ 42.94 km), as well as after a standardized 45-minute recovery break. Walking sessions were strictly time-matched to the individual marathon duration, with assessments performed at identical time points. Study 2 (Ultramarathon): Ultramarathon runners completed races of 100 km, 160 km, or 230 km. Blood samples and questionnaires were obtained before (Pre) and after (Post) completion of each distance

One or two months later, participants returned for a second session at the same time of day. When possible, the timing was also aligned with the menstrual cycle phase of female participants. All other procedures were identical. They walked along the marathon route while wearing HR monitors, with an alarm signaling when to turn back to match identical individual time durations and timing of blood sampling for running and walking (see Fig. 1). Questionnaires, blood sampling, and runner’s high assessments were therefore conducted at the same time points in both conditions. The participants were tested in small groups ranging from three to seven and could walk or run in groups as they preferred. Blood collection occurred in Study 1 between 12 p.m. and 6 p.m.

### Study 2: ultramarathon study

#### Participants and design

During the 2024 TorTour de Ruhr^®^, participants ran distances of 100 km, 160 km, and 230 km in central Germany following the river Ruhr from its source in Winterberg to its confluence with the Rhine in Duisburg. This study was part of a multicenter collaboration between the University Hospital Ulm, the University Hospital Essen, and the German Sport University Cologne [28, 29]. Inclusion criteria were male and female endurance runners participating in the 100 km, 160 km, or 230 km races of the TorTour de Ruhr^®^ 2024, with no prior injuries and the ability to understand the study procedures and provide informed consent.

Exclusion criteria included nicotine use, intestinal diseases, blood clotting disorders, or the use of blood-thinning medications, acute or chronic vascular disorders, cardiovascular, metabolic, or autoimmune diseases, and individuals unwilling to provide consent. Runners were informed about the study upon registration. Individuals were thoroughly informed about the study’s objectives, procedures, and data usage. Written informed consent was obtained from all participants.

#### Procedures and measurements

The study involved both pre- and post-race assessments. Pre-race measurements were conducted either the evening before the 230 km race (5:00 p.m. to 8:00 p.m.) or beginning two and a half hours before the start of the 160 km race (from 3:30 p.m. to 5:30 p.m.) and the 100 km race (from 1:30 a.m. to 3:30 a.m.). Post-race assessments took place immediately at the finish line upon participants’ arrival, following the initial celebrations, beginning with blood collection (with a delay of approximately five minutes). As a result, the timing of assessments varied across participants and race conditions, reflecting the logistical constraints of real-world ultramarathon events.

Assessments were conducted in temporary field laboratory settings established at designated race locations. Pre-race measurements were performed in indoor facilities (e.g., sports club buildings) at multiple stations along the course, allowing for standardized data collection procedures. Post-race assessments were conducted immediately at the finish line in a designated area under a sheltered outdoor pavilion within the race environment.

While efforts were made to standardize procedures across locations, environmental conditions such as temperature, humidity, and weather exposure varied between assessment sites. Weather conditions during the competition were cool, with light rain and temperatures ranging from 9 °C to 19 °C. Humidity was approximately 65%.

Before the race, participants completed sociodemographic questionnaires, IPAQ, EDS-R, and VAS. After the TorTour de Ruhr^®^, participants completed VAS and a post-race questionnaire including questions about runner’s high experiences.

#### Blood sampling

Blood samples were taken from a forearm vein using a butterfly needle (Multifly Needle with Adapter 21 G, Sarstedt, Nümbrecht, Germany) without delay in Study 1 and Study 2. Stasis time was kept under 30 s to minimize the risk of hemolysis. Plasma was collected using 7.5 mL ethylenediaminetetraacetic acid (EDTA)-coated tubes (S-Monovette, Sarstedt, Nümbrecht, Germany), which were immediately transferred to a cooled centrifuge located in the same area. Samples were centrifuged (2000 × g, 10 min at 4 °C) and the resulting plasma was aliquoted and stored at − 80 °C until further analysis [30]. Additionally, a 2.5 mL EDTA tube was collected to assess hematocrit (Hct) levels.

#### Assessment of Hct levels

Hct levels were measured at the Laboratory of the Institute of Medical Psychology and Behavioral Immunobiology, University Hospital Essen, using an automated hematology analyzer (XP-300, Sysmex Corporation, Kobe, Japan).

#### eCB extraction and measurement

eCBs, PEA, and AA were extracted and analyzed from 100-µL plasma samples following established standardized protocols [2, 23, 31, 32]. Briefly, plasma was extracted by parallelized extraction in 96-well plates and qualitatively and quantitatively analyzed by liquid chromatography mass spectrometry/multiple reaction monitoring on QTRAP 5500 (AB Sciex, Framingham, MA, USA). Deuterated eCBs, PEA and AA were spiked in the samples immediately after sample thawing to enable absolute quantification and account for any variability that might occur during sample preparation and analysis. Quantification was performed using MultiQuant software and the obtained values were normalized to plasma volume.

### Statistical analyses

#### Marathon

Analyses were conducted in R (version 4.5.1) [33] using RStudio (version 2025.09.1) [34]. To account for plasma volume changes due to exercise-induced hemoconcentration or hemodilution, eCB concentrations were normalized using the following formula for percent change in plasma volume [35]:

$$\begin{array}{l}\%\:change\:in\:plasma\:volume\\ \quad=\left(\frac{100}{\left[100-Hct\:pre\right]}\right)\times\left(\frac{\left[Hct\:pre\:-\:Hct\:post\right]}{Hct\:post}\right)\times100\hfill\end{array}$$

Next, they were log-transformed to correct for heteroscedasticity. For comprehensibility, the Hct-adjusted but not log-transformed values are reported in the text and figures. Linear mixed-effects (LME) analyses were applied to evaluate effects of condition (-0.5: walking; 0.5: marathon) and time point (1 − 5, dummy-coded) on AEA, 2-AG, 1-AG, AA, and PEA, respectively. The lme4 (version 1.1–37) [36] and lmerTest libraries (version 3.1-3) [37] were used for LME modeling and to evaluate significance. Significance was evaluated using restricted maximum likelihood and p-values were computed using Satterthwaite approximation [38]. While a maximal fit for random effects was attempted [39], only the intercept and condition slope could be included due to singular fit issues. An outlier criterion based on model critique [40] was applied, excluding data points with standardized residuals exceeding ± 2.5. Outliers were also evaluated on the subject level using Cook’s distance [41] via the influence.ME library (version 0.9-9) [42] at a cut-off of 4/(n-p-1); however, none of the subjects exceeded the criterion and thus the full sample was analyzed. Note that three data points were missing due to data loss during shipping of the blood vials; LME analysis is particularly suitable for handling missing data [43]. The model equation for the eCBs was as follows:

$$\begin{array}{l}AEA/2-AG/1-AG/AA/PEA\sim{condition}\\\quad\times\,{time}\,point+(1+condition|subject)\hfill\end{array}$$

Significant interactions were followed up using estimated marginal means [44], implemented with the emmeans package (version 1.11.2-8). P-values were corrected using Holm correction and degrees of freedom were calculated using the Kenward-Roger method.

Analyses of euphoria, pain, and anxiety closely followed the analyses of the eCBs. While euphoria scores did not need adjustments, a weight to account for differences in variance between time points needed to be used for anxiety ratings, and pain ratings needed to be log-transformed to correct for heteroscedasticity (as log(x + 1) to resolve issues with zeros). When weights were applied, the containment method was used to calculate degrees of freedom in post-hoc comparisons.

#### Ultramarathon

Analysis of the eCBs followed the procedure described above for the marathon data. LME analyses were applied, examining the effect of condition (100 km, 160 km, 230 km, dummy-coded) and time point (-0.5: before; 0.5: after) on AEA, 2-AG, 1-AG, AA, and PEA, respectively. Only a random intercept per subject was included due to the model being unidentifiable when time point is included as a random slope. One subject (100 km) had to be excluded from the analyses due to missing data from before the ultramarathon, impeding Hct correction. One more subject had to be excluded for the analysis of 1-AG and 2-AG due to exceeding the Cook’s distance criterion. The model equation was as follows:

$$\begin{array}{l}AEA/2-AG/1-AG/AA/PEA\sim{condition}\\\times\,{time}\,point+\left(1\right|subject)\end{array}$$

Analyses of euphoria, pain, and anxiety closely followed the analyses of the eCBs. While euphoria scores required no adjustment, anxiety ratings were weighted to account for variance differences between time points, and pain ratings were log-transformed to correct for heteroscedasticity. Two subjects needed to be excluded for the analysis of euphoria and one subject needed to be excluded from the analysis of anxiety due to exceeding the Cook’s distance criterion.

## Results

### Study 1: marathon study

A total of 19 participants were enrolled (7 females, 12 males; see Additional File 1: Tables S1 and S2). The average age was 46.2 years (SD = 9.9) with a mean BMI of 23.7 kg/m² (SD = 2.9). On average, participants ran 47 km per week (SD = 18.4) and reported predominantly high physical activity levels (94% of participants), with only one participant showing moderate activity. The mean distance covered was 42.94 km (SD = 0.65 km) for the marathon and 21.17 km (SD = 4.10 km) for the walking condition. One subject walked an exceptionally long distance during the walking condition (38.1 km). All participants were classified as *“non-dependent but symptomatic”* in the EDS-R.

### HR and pace during running and walking

**Fig. 2:**
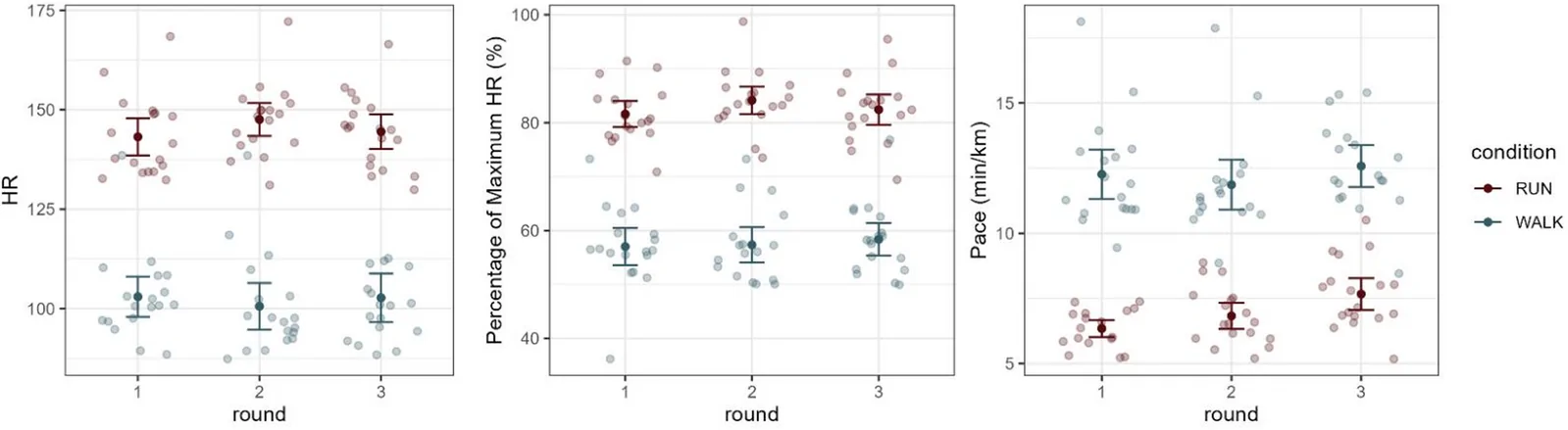
Heart rate, relative heart rate, and pace during marathon running and time-matched walking around Lake Baldeney, Essen (*n* = 19). Running (RUN, red) and walking (WALK, blue) conditions were compared within the same cohort. Heart rate (HR), relative HR expressed as percentage of age-predicted maximum HR (%HRmax), and pace were assessed across distance segments and conditions. HRmax was estimated using the Tanaka formula (HRmax = 208 − 0.7 × age). Data are shown as means ± SE; small dots represent individual values. Across all distance segments, HR was consistently higher during running than during walking. One participant deviated from the walking protocol requiring smooth, low-intensity walking and instead walked at high intensity, maintaining an HR of approximately 140 bpm, which was only slightly lower than the individual HR during running (approximately 154 bpm); this protocol deviation is visible in the elevated blue data points within the walking condition

On average, participants had an HR of 145.08 bpm (SE = 1.29) in the marathon and 102.09 bpm (SE = 1.65) in the walking condition. The percentage of maximum HR was calculated by the formula of Tanaka et al. (HRmax = 208 − 0.7 × age; [45]). On average, participants reached 82.71% of HRmax (SE = 0.76) during RUN compared with 57.59% of HRmax (SE = 0.94) during WALK. Mean pace was 6.95 min/km (SE = 0.16) during RUN and 12.24 min/km (SE = 0.26) during WALK. HR, percentage of HRmax, and pace across distance segments and conditions are presented in Fig. 2.

### Study 2: ultramarathon

A total of 36 ultramarathoners (13 females, 23 males) were included in the final analysis (230 km: *n* = 13; 160 km: *n* = 7; 100 km: *n* = 16; see Additional File 1: Table S3). Mean BMI was 23.5 kg/m² (SD = 2.4), and the average age was 50.2 ± 8.6 years. Participants had extensive endurance experience, completing on average 57 marathons and 41 ultramarathons. Weekly running distance averaged 71 ± 27 km per week, which was highest in the 230 km group. Most athletes were classified as “highly active” on the IPAQ, with moderate daily sitting times of around five to six hours. According to the EDS-R, most athletes were classified as *“non-dependent but symptomatic”*, while none were classified as at risk for exercise dependence.

### eCB responses to marathon, walking, and ultramarathon running

Circulating eCB responses were dominated by robust and temporally structured changes in AEA and 2-AG. During the marathon, AEA concentrations were significantly higher than during walking, with a pronounced condition × time point interaction (Table 1). While baseline levels did not differ between conditions, AEA increased progressively during marathon running, showing a trend toward higher concentrations than during walking after the first lap and reaching clear significance from the mid-race time point onward. Elevated AEA levels persisted at race completion and remained significantly higher than walking values 45 min post-exercise. In contrast, 2-AG exhibited a distinct biphasic pattern during the marathon (Table 1). Early during exercise, 2-AG levels were higher in the walking condition, whereas this pattern reversed during the later stages of the marathon and into early recovery, with significantly higher 2-AG concentrations observed following prolonged running. This marked condition-dependent crossover resulted in an interaction between condition and time point.

Ultramarathon running was likewise associated with pronounced alterations in AEA and 2-AG (Table 2). Across all ultramarathon distances, AEA levels increased substantially from pre- to post-race, reflecting a strong and consistent time effect independent of distance category. For 2-AG, post-race concentrations were also significantly elevated, with additional distance-related differences indicating higher levels following shorter compared with longer ultramarathon distances, although interaction effects did not reach statistical significance. Changes in other eCB-related lipids, including 1-AG, AA, and PEA, displayed analyte-specific time- and distance-dependent patterns but were less consistent across conditions and time points.

**Fig. 3:**
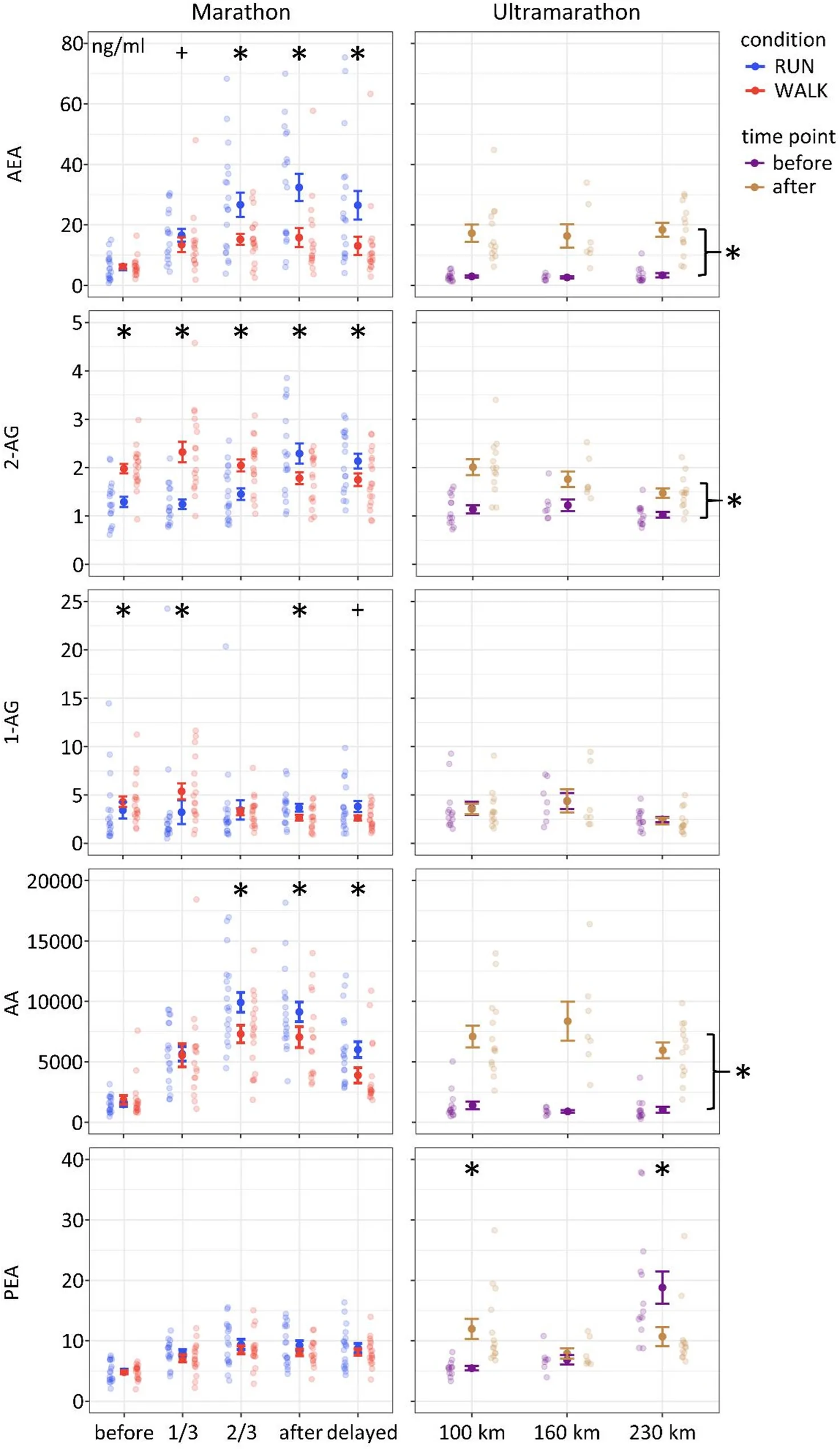
Endocannabinoid responses during marathon running (*n* = 19, Lake Baldeney, Essen) and ultramarathon competition (*n* = 36, TTdR). Circulating concentrations in ng/mL of eCBs and related lipids measured during a marathon (left panels) and ultramarathon competition (right panels). During the marathon, blood samples were collected before exercise (0 km), after each completed lap (14 km; 28 km), immediately after the run (≈ 42.94 km), and 45 min post-exercise, comparing running (RUN) and walking (WALK) conditions. During ultramarathons, samples were obtained before and after completion of 100-km, 160-km, and 230-km distances. Individual data points represent single participants; error bars indicate means ± standard error of the mean. Asterisks indicate significant interaction effects (*p* < .05), reflecting differences between RUN and WALK at specific marathon time points or between before and after within a given ultramarathon distance, while crosses (+) denote trend-level effects (0.05 ≤ *p* < .10). Brackets with asterisks denote significant main effects of time (before vs. after) across all ultramarathon distances. Main effects of distance are not shown for clarity. *AEA*,* anandamide (N-arachidonoylethanolamine); 2-AG*,* 2-arachidonoylglycerol; 1-AG*,* 1-arachidonoylglycerol; AA*,* arachidonic acid; PEA*,* palmitoylethanolamide; RUN*,* running; WALK*,* walking*

Model fit indices supported the robustness of these findings, with marginal/conditional R² values of 0.39/0.86 for AEA and 0.30/0.80 for 2-AG in the marathon study, and 0.73/0.78 for AEA and 0.49/0.66 for 2-AG in the ultramarathon study. The results are summarized in Tables 1 and 2 and illustrated in Fig. 3; all time-resolved concentrations, estimated marginal means, and post-hoc contrasts are provided in full in the Additional File 1: Tables S5–S14).

**Table 1:** Endocannabinoid and related lipid responses during marathon vs. walking around Lake Baldeney (time-matched conditions)

Analyte	Main Effect Condition	Main Effect Time	Condition × Time Interaction	Direction of Effects (post-hoc)
AEA	F(1, 17.94) = 6.98, *p* = 0.017	F(4, 137.34) = 91.33, *p* < 0.001	F(4, 137.33) = 12.77, *p* < 0.001	No baseline difference; higher during marathon from 14 km onward, persisting post-exercise
2-AG	F(1, 17.15) = 17.05, *p* < 0.001	F(4, 134.31) = 8.70, *p* < 0.001	F(4, 134.26) = 37.00, *p* < 0.001	Higher during walking early; reversed later with higher marathon levels at 42 km and post-exercise
1-AG	F(1, 17.94) = 1.77, *p* = 0.200	F(4, 138.37) = 0.59, *p* = 0.671	F(4, 138.37) = 19.51, *p* < 0.001	Initially lower during marathon, plateau mid-race, higher during marathon at later time points
AA	F(1, 18.17) = 6.92, *p* = 0.017	F(4, 138.00) = 130.85, *p* < 0.001	F(4, 137.97) = 4.29, *p* = 0.003	No early differences; higher during marathon from 28 km through post-exercise
PEA	F(1, 17.87) = 0.97, *p* = 0.339	F(4, 135.38) = 63.08, *p* < 0.001	F(4, 135.40) = 0.94, *p* = 0.442	Progressive increase over time, independent of condition

Shown are main effects of condition (marathon vs. walking), time point, and their interaction derived from linear mixed-effects models, as well as the direction of significant post-hoc contrasts across time points. Running and walking conditions were strictly time-matched, and blood sampling was performed at identical time points

*AEA* anandamide (N-arachidonoylethanolamine), 2-*AG* 2-arachidonoylglycerol; 1-*AG* 1-arachidonoylglycerol; *AA* arachidonic acid; *PEA* palmitoylethanolamide

**Table 2:** Endocannabinoid and related lipid responses to ultramarathon running (TTdR)

Analyte	Main Effect Condition (distance)	Main Effect Time (pre vs. post)	Condition × Time Interaction	Direction of Effects (post-hoc)
AEA	F(2, 31.13) = 0.25, *p* = 0.783	F(1, 30.90) = 201.94, *p* < 0.001	F(2, 30.95) = 0.02, *p* = 0.979	Marked increase after ultramarathon across all distances
2-AG	F(2, 30) = 3.33, *p* = 0.049	F(1, 30) = 67.03, *p* < 0.001	F(2, 30) = 2.00, *p* = 0.153	Higher post-race across distances; trend for higher levels at 100 km vs. 230 km
1-AG	F(2, 30) = 4.99, *p* = .013	F(1, 30) = 0.22, *p* = 0.643	F(2, 30) = 0.14, *p* = 0.872	Lower levels at 230 km compared with 100 km and 160 km; no time effect
AA	F(2, 31) = 1.10, *p* = 0.354	F(1, 31) = 251.46, *p* < 0.001	F(2, 31) = 0.88, *p* = 0.424	Strong increase after ultramarathon independent of distance
PEA	F(2, 30.48) = 16.17, *p* < .001	F(1, 30.27) = 1.25, *p* = 0.273	F(2, 30.37) = 18.82, *p* < 0.001	Higher levels at 230 km; post-race increase at 100 km, no change at 160 km, decrease at 230 km

Shown are the main effects of distance (100 km, 160 km, 230 km), time point (pre vs. post-race), and their interaction, derived from linear mixed-effects models, as well as the direction of significant post-hoc contrasts. Blood samples were collected immediately before and after completion of each ultramarathon distance

*AEA* anandamide (N-arachidonoylethanolamine), 2-*AG* 2-arachidonoylglycerol; 1-*AG* 1-arachidonoylglycerol; *AA* arachidonic acid; *PEA*, palmitoylethanolamide

### Psychological outcomes during marathon, walking and ultramarathon

**Fig. 4:**
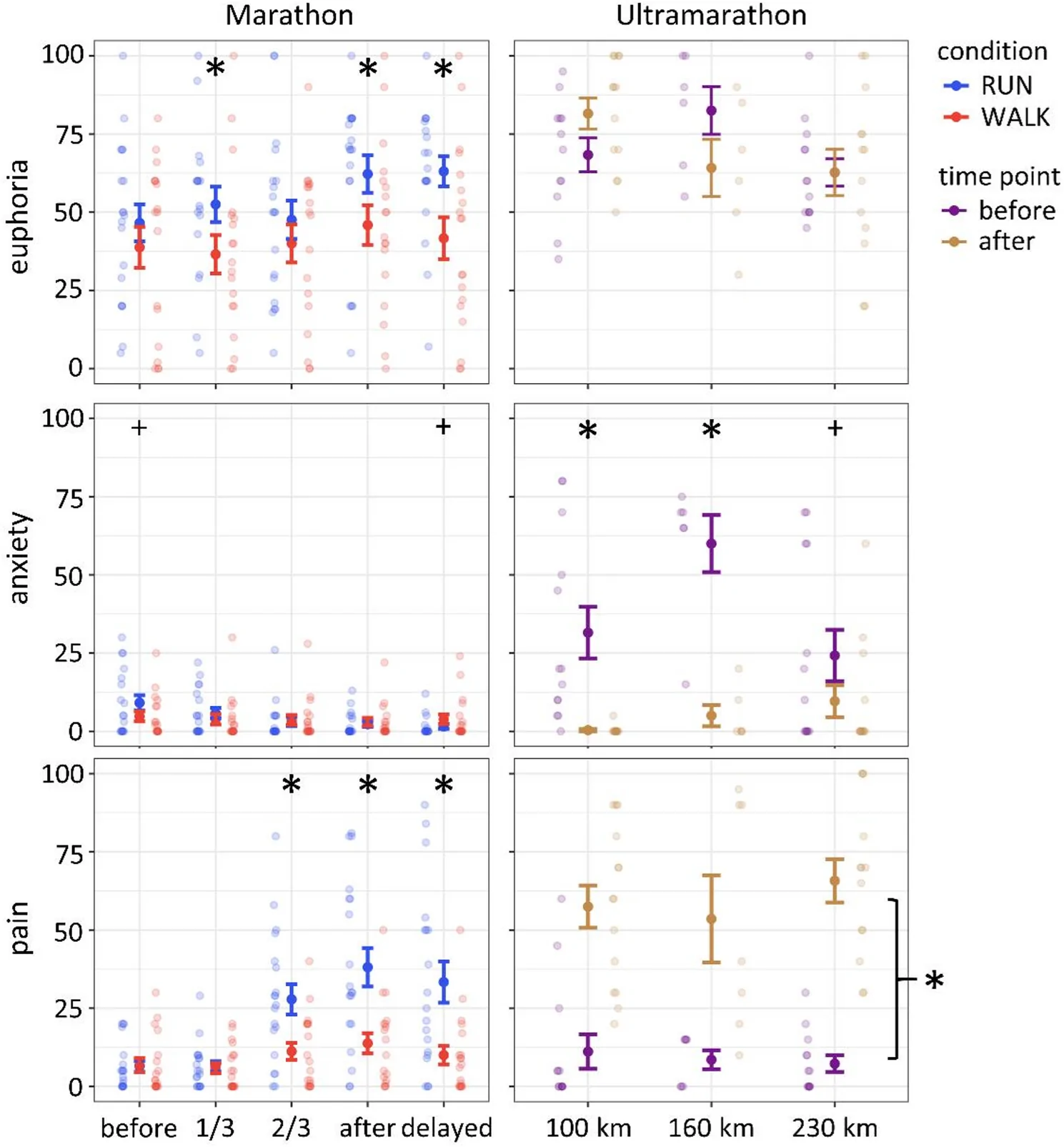
Psychological outcomes during marathon and ultramarathon running. Shown are subjective psychological measures (euphoria, anxiety, and pain) assessed during a marathon (left panels) and ultramarathons (right panels). During the marathon (*n* = 19), assessments were performed before exercise (0 km), after each completed lap (14 km; 28 km), immediately after the run (≈ 42.94 km), and 45 min post-exercise, comparing running (RUN) and walking (WALK) conditions. Ultramarathon data were obtained from 36 participants during the TorTour de Ruhr^®^ competition and assessed before and after completion of 100 km, 160 km, and 230 km distances. Individual data points represent single participants; error bars indicate means ± standard error of the mean. Overall, running was associated with higher euphoria and lower anxiety, while pain increased with prolonged distance, particularly during ultramarathon events. Asterisks indicate significant interaction effects (*p* < .05), reflecting differences between RUN and WALK at specific marathon time points or between before and after within a given ultramarathon distance, while crosses (+) denote trend-level effects (0.05 ≤ *p* < .10). Brackets with asterisks denote significant main effects of time (before vs. after) across all ultramarathon distances. Main effects of distance are not shown for clarity

Psychological responses differed between marathon and walking conditions and across ultramarathon distances (Tables 3 and 4; Fig. 4). During the marathon, euphoria and pain ratings were significantly higher compared with walking and changed over time, with condition × time interactions indicating more pronounced differences during the later stages of exercise. Anxiety showed a significant time-dependent modulation, with lower ratings toward the end of the marathon compared with walking. Under normal running conditions, runner’s high was reported as frequent by four participants, rare by five, or very rare by six; no participant reported always experiencing a runner’s high, while three reported never experiencing it. Retrospectively, in the marathon condition, three of 19 participants reported having experienced a runner’s high, whereas eight reported none and eight were unsure; during walking, only one participant reported a runner’s high, while the majority reported none. Pain was frequently reported during the marathon (11 of 19 participants) but was uncommon during walking (three of 19). Anxiety was rarely reported, with only one participant indicating anxiety during the marathon and none during walking (see Additional File 1: Table S2).

In the ultramarathon cohort, pain increased markedly from pre- to post-race across all distances, whereas anxiety decreased after completion of the race, with distance-dependent effects that were more pronounced at 100 km and 160 km than at 230 km. Euphoria did not show significant changes during ultramarathon running. All individual ratings and time-resolved values are provided in the Additional File 1: Tables S15–S20.

The majority (24 of 36) reported having experienced a runner’s high before, while only one had never experienced it (see Additional File 1: Table S4). During regular training, most described this feeling as occurring occasionally to frequently. During the race itself, 12 participants reported having experienced a runner’s high, whereas 19 did not. Pain was almost universal, with 29 of 36 runners reported pain during the race, whereas five reported no pain and eight reported the use of pain medication, particularly in the longest distance group. Anxiety during running was uncommon, reported by only five participants.

**Table 3:** Psychological outcomes during marathon and walking

Outcome	Effect	Statistic	*p*-value	Direction
Euphoria	Condition	F(1, 18.02) = 9.07	0.007	Marathon > Walking
	Time point	F(4, 140.36) = 4.52	0.002	Varies over time
	Condition × Time	F(4, 140.36) = 2.88	0.025	TP2, TP4, TP5
Pain	Condition	F(1, 17.93) = 10.54	0.004	Marathon > Walking
	Time point	F(4, 142.18) = 17.66	< 0.001	Increases over time
	Condition × Time	F(4, 142.18) = 3.03	0.020	TP3–TP5
Anxiety	Condition	F(1, 156) = 1.90	0.170	n.s.
	Time point	F(4, 156) = 5.02	< 0.001	Changes over time
	Condition × Time	F(4, 156) = 3.94	0.005	Lower post-marathon

Mixed-effects model results for euphoria, pain, and anxiety assessed repeatedly before, during, and after marathon running and a matched walking condition. Reported are main effects of condition and time point, as well as their interaction. Direction indicates the condition or time points at which significant differences were observed

TP = time point; n.s. = not significant

**Table 4:** Psychological outcomes during ultramarathon running (TTdR competition)

Outcome	Effect	Statistic	*p*-value	Direction
Euphoria	Condition	F(2, 30.79) = 2.51	0.098	Trend (100–160 km > 230 km)
	Time point	F(1, 30.34) = 0.10	0.749	n.s.
	Condition × Time	F(2, 30.63) = 2.57	0.093	Trend
Pain	Condition	F(2, 30.61) = 0.23	0.793	n.s.
	Time point	F(1, 29.46) = 96.95	< 0.001	Post > Pre
	Condition × Time	F(2, 29.68) = 0.80	0.460	n.s.
Anxiety	Condition	F(2, 32) = 1.66	0.207	n.s.
	Time point	F(1, 27) = 30.75	< 0.001	Post < Pre
	Condition × Time	F(2, 27) = 3.98	0.031	100 km & 160 km > 230 km

Results of mixed-effects models examining euphoria, pain, and anxiety before and after ultramarathon running across different race distances (100, 160, and 230 km). Reported are main effects of distance (condition), time point (pre vs. post), and their interaction. The direction column indicates the comparison underlying significant or trend-level effects. Note: Distance refers to ultramarathon length (100, 160, and 230 km)

n.s. = not significant

### Baseline differences between men and women (both marathon and ultramarathon)

There were no differences in baseline eCBs between men and women across the marathon and ultramarathon studies, all *p* ≥ .449. Sex also did not explain additional variance when added to the LME analyses as an additional factor, indicating that eCB progression did not differ between men and women, all *p* ≥ .075, except for AA in the marathon study (see Additional File 1: Table S21 and Figure S1).

### Differences in eCBs after the run between marathon and ultramarathon

In an additional exploratory analysis, we compared whether eCB levels immediately after a marathon differed from those after an ultramarathon. AEA, t(21.56) = 3.21, *p* = .004, 2-AG, t(23.34) = 2.47, *p* = .02, and AA, t(33.04) = 2.25, *p* = .031 were higher after the marathon than after ultramarathon running (see Table 5). There were no significant differences for 1-AG, t(41.9) = 0.92, *p* = .361, and PEA, t(49.13) = 1.13, *p* = .263.

**Table 5:** Means and standard deviations of eCBs after the marathon and ultramarathon

	*Marathon*	*Ultramarathon*
AEA	32.38 (19.00)	17.07 (9.46)
2-AG	2.29 (0.88)	1.73 (0.52)
AA	9129.83 (3440.10)	6916.33 (3248.20)

Post-exercise concentrations of eCBs and related lipids for marathon and ultramarathon runners. Values represent means with standard deviations

eCBs, endocannabinoids; *AEA* anandamide; 2-*AG* 2-arachidonoylglycerol; *AA* arachidonic acid

Note: n_Marathon_ = 19, n_Ultramarathon_ = 36

## Discussion

Our study shows eCB dynamics across marathon and ultramarathon running and affective responses during prolonged endurance exercise. Across both the marathon–walking design and the ultramarathon setting, we observed robust increases in several eCBs, especially AEA, which continued to rise beyond the first hour of exercise. In parallel, prolonged running was accompanied by acute affective changes, including higher euphoria ratings in the marathon study and reduced anxiety after prolonged running.

### eCB responses to walking, marathon and ultramarathon running

eCBs are produced on demand from AA derived from cell membrane phospholipids, particularly in the brain and skeletal muscle [46]. Thus, a sustained reservoir for eCB production exists, and we showed that this reservoir is sufficient to sustain eCB synthesis for more than 24 h of endurance running, consistent with the hypothesis that the eCB system may contribute to adaptations relevant to long-distance running [47].

Furthermore, we showed that eCB levels remained elevated even 45 min after a marathon distance. Previous studies suggested that eCB levels decline rapidly following endurance exercise [10, 25]. Here, we found that after a marathon eCB levels declined only gradually over the 45 min following the run. Thus, these eCBs may still act as neuromodulators with potential post-exercise effects (such as sedation).

In the marathon study, AEA increased markedly with distance and was consistently higher during running compared with walking, especially beyond 28 km and up to 45 min after race completion. In the current literature, AEA elevations during endurance sports are a robust finding [4, 10]. Because interruptions were minimized to allow blood sampling, this study showed a continuous rise in AEA during running.

On the other hand, 2-AG concentrations increased during the later stages of marathon running, during the recovery break, and within five minutes after the ultramarathons. The pattern is consistent with the broader literature, which shows more heterogeneous results for 2-AG compared with the more robust findings for AEA [4, 10, 24]. In our data, the elevation of 2-AG was only significant after running, leading us to hypothesize that AEA and 2-AG may support different psychological and physiological processes during endurance exercise, although no definitive mechanism can be inferred from our data. Given that AEA and 2-AG are synthesized via distinct enzymatic pathways (NAPE-PLD and DAGL, respectively), differences in their temporal dynamics are not unexpected.

In contrast, PEA showed a time-dependent effect but no condition-dependent effect. During the ultramarathon, PEA decreased after 230 km, increased after 100 km, and showed no significant change after 160 km. One possible explanation may relate to differences in nutritional status or timing of sample collection, as the 100 km race began in the early morning. Compared with AEA, increases in PEA have been reported less consistently in the literature. In a systematic review, only four out of ten studies observed elevations in PEA during endurance exercise (in contrast: AEA increases in 14 of 17 studies [10]). Notably, AEA and PEA, despite sharing common degradation pathways via FAAH, exhibited differing patterns in the present study, suggesting that regulatory mechanisms beyond enzymatic degradation may contribute to their dynamics during endurance exercise.

In an additional exploratory analysis, post-exercise eCB levels were higher following the marathon compared with the ultramarathon conditions across all distances. This finding is notable, as it suggests that factors beyond exercise duration may influence eCB responses. One possible explanation is the difference in relative exercise intensity. Marathon running is typically performed at a higher intensity, often closer to the anaerobic threshold, whereas ultramarathon running is characterized by substantially lower, more sustainable intensities. Given that previous research has linked eCB release to moderate-to-high intensity exercise, the lower eCB levels observed after ultramarathon running may reflect reduced intensity despite the longer duration [9]. This interpretation is in line with our findings from the walking condition, where lower-intensity activity was also associated with smaller increases in eCB levels. In addition, cumulative fatigue, energy depletion, and pacing strategies during ultramarathon events may further attenuate eCB responses. Together, these findings suggest that relative exercise intensity may be an important determinant of eCB dynamics, potentially exceeding the influence of exercise duration alone. However, given the exploratory nature of this comparison and the lack of direct intensity standardization, these interpretations should be considered with caution.

Another explanation for the lower 2-AG levels observed after 14 km and 28 km of running, compared with studies reporting elevated 2-AG levels after shorter exercise bouts, may be that well-trained individuals often exhibit reduced baseline eCB levels [10]. Because the participants in our study were highly trained runners (47–71 km running per week), baseline levels may have been comparatively low. This raises two possible hypotheses: first, endurance athletes may need to run longer distances or at higher intensities to elicit 2-AG and AEA responses comparable to those observed in less-trained individuals; second, they may regulate the production of 2-AG and AEA more precisely in an activity-dependent, *“on-demand”* manner. Our findings are more consistent with the first hypothesis, as 2-AG levels increased only gradually during the early phase of the run, particularly when compared with studies involving less-trained individuals and shorter exercise durations [23, 48–50].

One ambitious participant did not adhere to the study protocol requiring steady, low-intensity walking around Lake Baldeney, but instead walked at a high intensity while maintaining an HR (≈ 140 bpm) slightly lower than that during running (≈ 154 bpm). This participant’s eCB levels were comparable to those observed under running conditions, suggesting that intensity rather than locomotor biomechanics may drive eCB elevation. This finding is consistent with our earlier results and the study by Feuerecker et al. in which intense alpine hiking produced elevated eCB levels [23, 51].

### Psychological responses alongside eCB changes

On the psychological level, euphoria and pain ratings were higher during marathon running than during walking at several time points, while state anxiety showed a trend-level reduction toward the end of the run. Euphoria showed a clear condition × time interaction, with higher ratings in the marathon condition particularly at intermediate and late time points.

At 28 km, euphoria levels during the marathon did not significantly differ from those during walking, which were similar to baseline levels. This running phase typically coincides with the metabolic shift toward fat oxidation and the feeling of *“hitting the wall”* is commonly reported [52, 53].

Pain ratings followed a different pattern. Perceived exercise-related pain increased markedly over time and was consistently higher in the marathon than in the walking condition at later time points. Pain ratings did not increase after 14 km of running, whereas a significant elevation became apparent only at the 28 km time point. These findings should be interpreted cautiously, as subjective exercise-related pain differs from exercise-induced hypoalgesia. The latter refers to an increased pain threshold in response to experimentally induced stimuli (e.g., pressure or temperature) and does not directly reflect the perception of tissue stress or microtrauma during exercise. One possible explanation is that during a marathon, pain resulting from cumulative physical strain may exceed the capacity of endogenous analgesic mechanisms to fully suppress its perception. Moreover, five participants reported retrospectively that they did not feel any pain during an ultramarathon. This observation suggests that exercise can still induce pronounced hypoalgesic effects, even under extreme physiological load.

Anxiety showed modest but meaningful changes. In the marathon, baseline state anxiety was slightly higher in the running condition and decreased toward the end of the run, whereas walking showed a flatter development. In the ultramarathon, anxiety was significantly reduced after the race, particularly in the 100 and 160 km groups, with a weaker effect in the 230 km group. Importantly, most of the participants were unfamiliar with scientific study procedures, including repeated blood sampling, and ultramarathon runners often competed on unfamiliar routes in the dark, frequently running completely alone. In addition, the marathon running condition was conducted before the walking condition, which may have contributed to elevated baseline anxiety due to anticipatory uncertainty. Despite these challenging conditions, most runners retrospectively reported having felt no anxiety during the runs (marathon: 18/19; ultramarathon: 29/36), highlighting anxiolytic effects as a robust psychological change. Overall, these patterns are in line with the view that eCB signaling contributes to the anxiolytic and calming components of long-term running, but also that this relationship is not linear and is strongly influenced by context and duration.

Given these psychological changes, it is not surprising that the EDS-R identified a substantial number of athletes as *“non-dependent but symptomatic”* (marathon: 19/19; ultramarathon: 31/36). Multiple studies have identified addiction-like behaviors in endurance athletes [54–56]. These include craving, dysphoric withdrawal symptoms during training interruptions, and a compulsive urge to continue exercising despite adverse consequences. One possible mechanism for exercise dependence might be that athletes gradually increase training volume to achieve the same rewarding effects through eCBs as baseline levels are significantly lower [10]. As a result, some individuals may continue running even when injured, exhausted, or when training begins to interfere with family life and social responsibilities.

### Strengths, limitations, and future directions

A major strength of this field study is the combination of a within-subject marathon-walking design with an ultramarathon cohort spanning three distances. The repeated blood sampling, comprehensive affective measures, and rigorous mixed-effects modeling provide a detailed characterization of eCB dynamics in combination with psychological changes.

We recognize several limitations in our study. Using walking as a comparison condition may have reduced observable differences in eCB levels. A more sedentary control, such as sitting, could have provided a stronger contrast. However, we were also interested in exploring the positive psychological effects of walking in nature and our earlier research showed an increase in eCBs through walking on a treadmill [23], which could be replicated in the present field-based setting. At the same time, this approach introduces a limitation, as walking represents a lower-intensity physiological condition rather than a neutral baseline. Therefore, differences between conditions should be interpreted as reflecting relative differences in exercise intensity rather than the presence versus absence of physiological activation.

Allowing participants to run and walk under preferred conditions introduced variability but ensured ecological validity. As a result, physiological load likely differed between participants, which may have contributed to variability in eCB responses. However, our primary aim was to preserve conditions that more closely reflect real-world running and walking behavior, rather than impose strictly controlled laboratory settings. In particular, differences in pacing strategies during the marathon may have led to heterogeneous exercise intensities across participants, which could not be accounted for in the present analyses. In addition, in both studies, time-of-day and contextual differences between sessions (e.g., nutritional status, rest, and anticipatory stress) were not fully controlled and may have influenced eCB levels, representing a potential source of bias in the interpretation of the findings.

A further limitation is the use of single-item VAS to assess complex psychological constructs such as euphoria and anxiety. While VAS is well validated for the assessment of pain [57], their application to multidimensional affective states is less established and should therefore be interpreted as an approximate rather than a comprehensive assessment. This is particularly relevant given that the runner’s high is considered a multidimensional phenomenon, encompassing affective, cognitive, and perceptual components. However, the use of brief VAS measures allowed for rapid data collection in a field setting, minimizing disruption of the exercise protocol and enabling the assessment of acute, in-exercise changes rather than delayed post-exercise effects.

Several methodological limitations should be considered, including the small sample sizes, heterogeneity between the marathon and ultramarathon study designs, the limited number of measured eCB-related lipids, and the absence of longer-term follow-up samples, all of which restrict direct comparability between studies and limit mechanistic interpretation.

Furthermore, the competitive nature of the TorTour de Ruhr^®^ may have driven participants closer to their physical limits, potentially hindering the development of a runner’s high due to catecholamine-driven stress responses [58]. Due to the race setting, it was not possible to obtain samples during the competition, which limits the comparability between Study 1 and Study 2. Lastly, because space for equipment was limited at the 230 km starting area, pre-run samples were collected the evening before the race and may have differed from baseline levels at the beginning of the race.

To date, nearly all studies investigating eCB signaling during running in humans have relied on peripheral blood sampling to examine the influence of eCBs on the runner’s high. A study in mice showed that anxiolysis depends on intact CB1 receptors on forebrain GABAergic neurons, while pain reduction is mediated by the activation of peripheral CB1 and CB2 receptors [2]. Future research should aim to explore eCB signaling directly in humans. For example, using PET imaging could help bridge this knowledge gap as some radiotracers have already been tested in humans [59–61]. Additionally, integrating multiple neurochemicals known to play a crucial role in the runner’s high, such as eCBs, leptin, BDNF, cortisol, oxytocin, serotonin, dopamine, and noradrenaline, would provide a more comprehensive understanding of this phenomenon.

## Conclusions

In conclusion, our findings show robust and time-dependent changes in circulating eCB levels during prolonged endurance exercise. AEA increased continuously during running whereas 2-AG showed a delayed rise. In parallel, the marathon study showed higher euphoria and lower anxiety ratings during running than during walking, particularly at later time points. The persistent elevation of eCBs after 45 min of recovery further highlights their relevance for post-exercise mood changes and calming effects. Overall, our findings suggest that changes in circulating eCB concentrations may represent an important physiological feature of prolonged endurance exercise in humans and occur alongside affective changes to potentially promote long-distance running.

## Supplementary Information

Below is the link to the electronic supplementary material.


Supplementary Material 1: Additional File 1: Tables S1–S21 and Figure S1


## Data Availability

The anonymized individual-level biochemical datasets supporting the eCB analyses are available in the Zenodo repository [62]. Additional data not included in the public dataset are not publicly available because their combination may contain potentially identifiable information and because of ethical and data protection restrictions related to the field-based study design. Access to these data may be requested from the corresponding author, Michael Siebers, for scientifically justified purposes. Requests will be reviewed by the authors and, where required, the responsible ethics committee. A response will be provided within four weeks. Data will be shared only after approval and completion of a data use agreement restricting use to the approved research purpose.

## Abbreviations

AA: arachidonic acid
AEA: anandamide (N–arachidonoylethanolamine)
AG: arachidonoylglycerol
BMI: body mass index
CB1: cannabinoid receptor 1
CB2: cannabinoid receptor 2
eCB: endocannabinoid
EDS-R: Exercise Dependence Scale–Revised
EDTA: ethylenediaminetetraacetic acid
FAAH: fatty acid amide hydrolase
GPS: global positioning system
Hct: hematocrit
HR: heart rate
IPAQ: International Physical Activity Questionnaire
LME: linear mixed–effects model
MAGL: monoacylglycerol lipase
PEA: palmitoylethanolamide
RUN: running
SD: standard deviation
SE: standard error
TTdR: TorTour de Ruhr^®^
VAS: visual analog scale
WALK: walking

## References

[1] Lieberman DE, Bramble DM, Raichlen DA, Shea JJ. The evolution of endurance running and the tyranny of ethnography: a reply to Pickering and Bunn (2007). J Hum Evol. 2007;53:439–42. 10.1016/j.jhevol.2007.07.002 17767947

[2] Fuss J, Steinle J, Bindila L, Auer MK, Kirchherr H, Lutz B, et al. A runner’s high depends on cannabinoid receptors in mice. Proc Natl Acad Sci U S A. 2015;112:13105–8. 10.1073/pnas.1514996112. 26438875 PMC4620874

[3] Dubreucq S, Koehl M, Abrous DN, Marsicano G, Chaouloff F. CB1 receptor deficiency decreases wheel-running activity: consequences on emotional behaviours and hippocampal neurogenesis. Exp Neurol. 2010;224:106–13. 10.1016/j.expneurol.2010.01.017. 20138171

[4] Desai S, Borg B, Cuttler C, Crombie KM, Rabinak CA, Hill MN, et al. A systematic review and meta-analysis on the effects of exercise on the endocannabinoid system. Cannabis Cannabinoid Res. 2022;7(4):388–408. 10.1089/can.2021.0113. 34870469 PMC9418357

[5] Dietrich A, McDaniel WF. Endocannabinoids and exercise. Br J Sports Med. 2004;38:536–41. 10.1136/bjsm.2004.011718. 15388533 PMC1724924

[6] Mechoulam R, Hanuš LO, Pertwee R, Howlett AC. Early phytocannabinoid chemistry to endocannabinoids and beyond. Nat Rev Neurosci. 2014;15:757–64. 10.1038/nrn3811. 25315390

[7] Baggelaar MP, Maccarrone M, van der Stelt M. 2-Arachidonoylglycerol: a signaling lipid with manifold actions in the brain. Prog Lipid Res. 2018;71:1–17. 10.1016/j.plipres.2018.05.002. 29751000

[8] Cristino L, Bisogno T, Di Marzo V. Cannabinoids and the expanded endocannabinoid system in neurological disorders. Nat Rev Neurol. 2020;16:9–29. 10.1038/s41582-019-0284-z. 31831863

[9] Raichlen DA, Foster AD, Seillier A, Giuffrida A, Gerdeman GL. Exercise-induced endocannabinoid signaling is modulated by intensity. Eur J Appl Physiol. 2013;113:869–75. 10.1007/s00421-012-2495-5. 22990628

[10] Siebers M, Biedermann SV, Fuss J. Do endocannabinoids cause the runner’s high? Evidence and open questions. Neuroscientist. 2023;29(3):352–69. 10.1177/10738584211069981. 35081831 PMC10159215

[11] Black J, Chesher GB, Starmer GA, Egger G. The painlessness of the long distance runner. Med J Aust. 1979;1:522–3. 10.5694/j.1326-5377.1979.tb119352.x. 470706

[12] Partin C. Runner’s high. JAMA. 1983;249:21. 10.1001/jama.1983.03330250017014. 6848779

[13] Dishman RK, O’Connor PJ. Lessons in exercise neurobiology: the case of endorphins. Ment Health Phys Act. 2009;2:4–9. 10.1016/j.mhpa.2009.01.002.

[14] Boecker H, Sprenger T, Spilker ME, Henriksen G, Koppenhoefer M, Wagner KJ, et al. The runner’s high: opioidergic mechanisms in the human brain. Cereb Cortex. 2008;18:2523–31. 10.1093/cercor/bhn013. 18296435

[15] Goldfarb AH. Exercise and endogenous opiates. In: Constantini N, Hackney AC, editors. Endocrinology of Physical Activity and Sport. Totowa, NJ: Humana; 2013. pp. 21–36. 10.1007/978-1-62703-314-5_2.

[16] Goldfarb AH, Hatfield BD, Armstrong D, Potts J. Plasma beta-endorphin concentration: response to intensity and duration of exercise. Med Sci Sports Exerc. 1990;22:241–4. 2141380

[17] Saanijoki T, Tuominen L, Tuulari JJ, Nummenmaa L, Arponen E, Kalliokoski K, et al. Opioid release after high-intensity interval training in healthy human subjects. Neuropsychopharmacology. 2018;43:246–54. 10.1038/npp.2017.148. 28722022 PMC5729560

[18] Saanijoki T, Nummenmaa L, Tuulari JJ, Tuominen L, Arponen E, Kalliokoski KK, et al. Aerobic exercise modulates anticipatory reward processing via the µ-opioid receptor system. Hum Brain Mapp. 2018;39:3972–83. 10.1002/hbm.24224. 29885086 PMC6866313

[19] Heyman E, Gamelin F-X, Goekint M, Piscitelli F, Roelands B, Leclair E, et al. Intense exercise increases circulating endocannabinoid and BDNF levels in humans: possible implications for reward and depression. Psychoneuroendocrinology. 2012;37:844–51. 10.1016/j.psyneuen.2011.09.017. 22029953

[20] Sparling PB, Giuffrida A, Piomelli D, Rosskopf L, Dietrich A. Exercise activates the endocannabinoid system. NeuroReport. 2003;14:2209–11. 10.1097/00001756-200312020-00015. 14625449

[21] Gasperi V, Ceci R, Tantimonaco M, Talamonti E, Battista N, Parisi A, et al. The fatty acid amide hydrolase in lymphocytes from sedentary and active subjects. Med Sci Sports Exerc. 2014;46:24–32. 10.1249/MSS.0b013e3182a10ce6. 23793235

[22] Matei D, Trofin D, Iordan DA, Onu I, Condurache I, Ionite C, et al. The endocannabinoid system and physical exercise. Int J Mol Sci. 2023;24:1989. 10.3390/ijms24031989. 36768332 PMC9916354

[23] Siebers M, Biedermann SV, Bindila L, Lutz B, Fuss J. Exercise-induced euphoria and anxiolysis do not depend on endogenous opioids in humans. Psychoneuroendocrinology. 2021;126:105173. 10.1016/j.psyneuen.2021.105173. 33582575

[24] Dalle S, Poffé C, Lauriks W, Robberechts R, Stalmans M, Terrasi R, et al. Circulating endocannabinoids are associated with mental alertness during ultra-endurance exercise. Cannabis Cannabinoid Res. 2025;10(2):200–6. 10.1089/can.2024.0169. 39729203

[25] Hillard CJ. Circulating endocannabinoids: from whence do they come and where are they going? Neuropsychopharmacology. 2018;43:155–72. 10.1038/npp.2017.130. 28653665 PMC5719092

[26] Craig CL, Marshall AL, Sjöström M, Bauman AE, Booth ML, Ainsworth BE, et al. International physical activity questionnaire: 12-country reliability and validity. Med Sci Sports Exerc. 2003;35:1381–95. 10.1249/01.MSS.0000078924.61453.FB. 12900694

[27] Hausenblas HA, Downs DS. How much is too much? The development and validation of the Exercise Dependence Scale. Psychol Health. 2002;17:387–404. 10.1080/0887044022000004894.

[28] Grau M, Bruns J, Stücher M, John L, Munk M, Siebers M, et al. Running on the edge: hematological responses to a non-stop ultramarathon with a focus on nitric oxide–mediated red blood cell deformability. Front Physiol. 2025;16:1715513. 10.3389/fphys.2025.1715513. 41357163 PMC12678145

[29] John L, Munk M, Bizjak R, Schulz SVW, Witzel J, Engler H, et al. Does distance matter? Metabolic and muscular challenges of a non-stop ultramarathon with sub-analysis depending on running distance. Nutrients. 2025;17:3801. 10.3390/nu17233801. 41374091 PMC12693733

[30] Hargreaves J, Ney L. Experimental and pre-analytical considerations of endocannabinoid quantification in human biofluids prior to mass spectrometric analysis. Targets. 2025;3:11. 10.3390/targets3010011.

[31] Post JM, Lerner R, Schwitter C, Lutz B, Lomazzo E, Bindila L. Lipidomics and transcriptomics in neurological diseases. J Vis Exp. 2022;181e59423. 10.3791/59423. 35377364

[32] Schwitter C, Lutz B, Bindila L. Extraction and simultaneous quantification of endocannabinoids and endocannabinoid-like lipids in biological tissues. In: Maccarrone M, editor. Endocannabinoid Signaling. New York, NY: Springer US; 2023. pp. 9–19. 10.1007/978-1-0716-2728-0_2. 36152174

[33] R Core Team. R: a language and environment for statistical computing. Vienna: R Foundation for Statistical Computing; 2025.

[34] Posit Team. RStudio: integrated development environment for R. Boston, MA: Posit Software, PBC; 2025.

[35] Van Beaumont W. Evaluation of hemoconcentration from hematocrit measurements. J Appl Physiol. 1972;32:712–3. 10.1152/jappl.1972.32.5.712. 5038863

[36] Bates D, Mächler M, Bolker B, Walker S. Fitting linear mixed-effects models using lme4. J Stat Softw. 2015;67:1–48. 10.18637/jss.v067.i01.

[37] Kuznetsova A, Brockhoff PB, Christensen RHB. lmerTest package: tests in linear mixed-effects models. J Stat Softw. 2017;82(13):1–26. 10.18637/jss.v082.i13.

[38] Luke SG. Evaluating significance in linear mixed-effects models. Behav Res Methods. 2017;49:1494–502. 10.3758/s13428-016-0809-y. 27620283

[39] Brauer M, Curtin JJ. Linear mixed-effects models and the analysis of nonindependent data: a unified framework to analyze categorical and continuous independent variables that vary within-subjects and/or within-items. Psychol Methods. 2018;23:389–411. 10.1037/met0000159. 29172609

[40] Baayen RH, Milin P. Analyzing reaction times. Int J Psychol Res (Medellin). 2010;3:12–28. 10.21500/20112084.807.

[41] Cook RD. Detection of influential observation in linear regression. Technometrics. 1977;19:15–8. 10.1080/00401706.1977.10489493.

[42] Nieuwenhuis R, te Grotenhuis M, Pelzer B. influence.ME: tools for detecting influential data in mixed-effects models. R J. 2012;4:38–47. 10.32614/RJ-2012-011.

[43] Gabrio A, Plumpton C, Banerjee S, Leurent B. Linear mixed models to handle missing at random data in trial-based economic evaluations. Health Econ. 2022;31:1276–87. 10.1002/hec.4510. 35368119 PMC9325521

[44] Searle SR, Speed FM, Milliken GA. Population marginal means in the linear model: an alternative to least squares means. Am Stat. 1980;34:216–21. 10.1080/00031305.1980.10483031.

[45] Tanaka H, Monahan KD, Seals DR. Age-predicted maximal heart rate revisited. J Am Coll Cardiol. 2001;37:153–6. 10.1016/S0735-1097(00)01054-8. 11153730

[46] Tallima H, El Ridi R. Arachidonic acid: physiological roles and potential health benefits—a review. J Adv Res. 2018;11:33–41. 10.1016/j.jare.2017.11.004. 30034874 PMC6052655

[47] Bramble DM, Lieberman DE. Endurance running and the evolution of Homo. Nature. 2004;432:345–52. 10.1038/nature03052. 15549097

[48] Brellenthin AG, Crombie KM, Hillard CJ, Koltyn KF. Endocannabinoid and mood responses to exercise in adults with varying activity levels. Med Sci Sports Exerc. 2017;49:1688–96. 10.1249/MSS.0000000000001276. 28319590

[49] Crombie KM, Cisler JM, Hillard CJ, Koltyn KF. Aerobic exercise reduces anxiety and fear ratings to threat and increases circulating endocannabinoids in women with and without PTSD. Ment Health Phys Act. 2021;20:100366. 10.1016/j.mhpa.2020.100366. 34149867 PMC8208522

[50] Crombie KM, Brellenthin AG, Hillard CJ, Koltyn KF. Psychobiological responses to aerobic exercise in individuals with posttraumatic stress disorder. J Trauma Stress. 2018;31:134–45. 10.1002/jts.22253. 29388710

[51] Feuerecker M, Hauer D, Toth R, Demetz F, Hölzl J, Thiel M, et al. Effects of exercise stress on the endocannabinoid system in humans under field conditions. Eur J Appl Physiol. 2012;112:2777–81. 10.1007/s00421-011-2237-0. 22101870

[52] Buman MP, Brewer BW, Cornelius AE, van Raalte JL, Petitpas AJ. Hitting the wall in the marathon: phenomenological characteristics and associations with expectancy, gender, and running history. Psychol Sport Exerc. 2008;9:177–90. 10.1016/j.psychsport.2007.03.003.

[53] Venhorst A, Micklewright DP, Noakes TD. Modelling perception-action coupling in the phenomenological experience of hitting the wall during long-distance running with exercise-induced muscle damage in highly trained runners. Sports Med Open. 2018;4:30. 10.1186/s40798-018-0144-1. 29987475 PMC6037658

[54] Antunes HKM, Leite GSF, Lee KS, Barreto AT, Santos RVTD, de Souza H. Exercise deprivation increases negative mood in exercise-addicted subjects and modifies their biochemical markers. Physiol Behav. 2016;156:182–90. 10.1016/j.physbeh.2016.01.028. 26812592

[55] Lukács A, Sasvári P, Varga B, Mayer K. Exercise addiction and its related factors in amateur runners. J Behav Addict. 2019;8:343–9. 10.1556/2006.8.2019.28. 31146551 PMC7044555

[56] Remilly M, Mauvieux B, Drigny J. Personality traits associated with the risk of exercise dependence in ultraendurance athletes: a cross-sectional study. Int J Environ Res Public Health. 2023;20:1042. 10.3390/ijerph20021042. 36673797 PMC9858902

[57] Hawker GA, Mian S, Kendzerska T, French M. Measures of adult pain: Visual Analog Scale for Pain (VAS Pain), Numeric Rating Scale for Pain (NRS Pain), McGill Pain Questionnaire (MPQ), Short-Form McGill Pain Questionnaire (SF‐MPQ), Chronic Pain Grade Scale (CPGS), Short Form‐36 Bodily Pain Scale (SF‐36 BPS), and Measure of Intermittent and Constant Osteoarthritis Pain (ICOAP). Arthritis Care Res (Hoboken). 2011;63(Suppl 11):S240–52. 10.1002/acr.20543. 22588748

[58] Karsai I, Nagy Z, Nagy T, Kocsor F, Láng A, Kátai E, et al. Physical exercise induces mental flow related to catecholamine levels in noncompetitive, but not competitive conditions in men. Sci Rep. 2023;13:14238. 10.1038/s41598-023-41518-2. 37648819 PMC10469213

[59] Burns HD, Van Laere K, Sanabria-Bohórquez S, Hamill TG, Bormans G, Eng WS, et al. [18F]MK-9470, a positron emission tomography (PET) tracer for *in vivo* human PET brain imaging of the cannabinoid-1 receptor. Proc Natl Acad Sci U S A. 2007;104:9800–5. 10.1073/pnas.0703472104. 17535893 PMC1877985

[60] Terry GE, Hirvonen J, Liow J-S, Zoghbi SS, Gladding R, Tauscher JT, et al. Imaging and quantitation of cannabinoid CB1 receptors in human and monkey brains using (18)F-labeled inverse agonist radioligands. J Nucl Med. 2010;51:112–20. 10.2967/jnumed.109.067074. 20008988 PMC2997525

[61] Normandin MD, Zheng M-Q, Lin K-S, Mason NS, Lin S-F, Ropchan J, et al. Imaging the cannabinoid CB1 receptor in humans with [11 C]OMAR: assessment of kinetic analysis methods, test-retest reproducibility, and gender differences. J Cereb Blood Flow Metab. 2015;35:1313–22. 10.1038/jcbfm.2015.46. 25833345 PMC4528005

[62] Siebers M, Huvermann D, Siebers C, Canales-Romero D, Florea-Ghile A, Keite L, John L, Munk M, Bizjak R, Witzel J, Schulz S, Kirsten J, Bindila L, Hinney A, Grau M, Bizjak DA, Engler H, Fuss J. Endocannabinoid Dynamics Across Marathon and Ultramarathon Running: Evidence from Two Field Studies. Zenodo. 2026. 10.5281/zenodo.20208759.

