# Supplementary material for: Endocannabinoid dynamics across marathon and ultramarathon running: evidence from two field studies
Source: BMC Med. 2026 Sep 1;24:469. doi: 10.1186/s12916-026-05186-z (PMC13531787; doi:10.1186/s12916-026-05186-z)
Supplement: Supplementary file 1 [file 12916_2026_5186_MOESM1_ESM.docx]

**Additional File 1**

**Endocannabinoid Dynamics Across Marathon and Ultramarathon Running: Evidence from Two Field Studies**

Michael Siebers^1,2*^, Dana Huvermann^1^, Christoph Siebers^1^, Deborah Canales-Romero^1^, Alexandra Florea-Ghile^2^, Lina Keite^1^, Lucas John^3^, Moritz Munk^3^, Roman Bizjak^3^, Jens Witzel^4^, Sebastian Schulz^3^, Johannes Kirsten^3^, Laura Bindila^5^, Anke Hinney^6^, Marijke Grau^7^, Daniel A Bizjak^3^, Harald Engler^8^, Johannes Fuss^1^

^1^Institute for Forensic Psychiatry and Sexual Research, Center for Translational Neuro- and Behavioral Sciences, University of Duisburg-Essen, Essen, Germany.

^2^Department of Psychiatry and Psychotherapy, LVR University Hospital Essen, Center for Translational Neuro- and Behavioral Sciences, University of Duisburg-Essen, Essen, Germany.

^3^Department of Internal Medicine, Division of Sports and Rehabilitation Medicine, University Hospital Ulm, Ulm, Germany.

^4^Department of Central IT, Division Applications and Databases, University of Zurich, Zurich, Switzerland.

^5^Clinical Lipidomics Unit, Institute of Physiological Chemistry, University Medical Center of the Johannes Gutenberg University Mainz, Mainz, Germany.

^6^Section of Molecular Genetics in Mental Disorders, Center for Translational Neuro- and Behavioral Sciences, Institute of Sex and Gender-Sensitive Medicine, University of Duisburg-Essen, Essen, Germany.

^7^Institute of Cardiovascular Research and Sports Medicine, Molecular and Cellular Sports Medicine, German Sport University Cologne, Cologne, Germany.

^8^Institute of Medical Psychology and Behavioral Immunobiology, Center for Translational Neuro- and Behavioral Sciences, University Hospital Essen, University of Duisburg-Essen, Essen, Germany.

*Corresponding author

Michael Siebers; Email: [Michael.Siebers@LVR.de](mailto:Michael.Siebers@LVR.de)

ORCID:

Michael Siebers: 0000-0002-1400-2176

**Table S1. Sociodemographic and Self-Reported Running Characteristics of Marathon Runners at Lake Baldeney 2024**

| *Participants (n)* | 19 (f=7, m=12) |
| --- | --- |
| *BMI (kg/m²)* | 23.68 (SD 2.92) |
| *Age* | 46.21 (SD 9.94) |
| *Weekly running distance (km)* | 47.05 (SD 18.36) |
| *Marathons*  *Participated*  *Finished* | 46.53  (SD 85.04)  (Min =1; Max =380)  (Median = 15)  45.11  (SD 85.22)  (Min = 1; Max = 370)  (Median = 15) |
| *Ultramarathons*  *Participated*  *Finished* | 15.95  (SD 30.89)  (Min = 0; Max = 130)  (Median = 3)  15.90  (SD 30.88)  (Min = 0; Max = 130)  (Median = 3) |
| *Physical activity levels*  *Low*  *Moderate*  *High*  *Sitting/day (min.)* | 0  1  18  412.89 (SD 135.49) |
| *Did you ever experience a runner's high before?*  *Yes*  *No*  *I do not know* | 12  2  5 |
| *How often do you experience a runner's high under normal running conditions?*  *Always*  *Frequently*  *Rarely*  *Very rarely*  *Never*  *No answer* | 0  4  5  6  3  1 |
| *EDS-R*  *At-risk for exercise dependence*  *Non-dependent but symptomatic*  *Non-dependent* | 0  19  0 |

Sociodemographic and running-related data of participants in the Lake Baldeney Marathon Study 2024. Values are means with standard deviations (SD) or absolute frequencies. BMI = Body Mass Index; IPAQ = International Physical Activity Questionnaire; EDS-R = Exercise Dependence Scale-Revised; NA = no answer, n = 19.

**Table S2. Runner’s High, Pain, and Anxiety During Marathon vs. Walking (Lake Baldeney 2024, n = 19)**

|  | Marathon | Walking |
| --- | --- | --- |
| *Did you experience a runner's high?*  *Yes*  *No*  *I do not know* | 3  8  8 | 1  13  5 |
| *Did you experience pain during running/walking?*  *Yes*  *No*  *I do not know* | 11  7  1 | 3  14  2 |
| *Did you experience anxiety during running/walking?*  *Yes*  *No*  *I do not know* | 1  18  0 | 0  19  0 |

Self-reported experiences of runner’s high, pain, and anxiety during the marathon and walking conditions of the Lake Baldeney Study 2024. Values represent absolute frequencies. NA = no answer.

**Table S3**: **Sociodemographic Characteristics of Ultramarathon Participants in the TorTour de Ruhr® (TTdR) 2024**

|  | TTdR  230 km | TTdR  160 km | TTdR  100 km | Total  values |
| --- | --- | --- | --- | --- |
| *Participants (n)* | 13  (f=3, m=10) | 7  (f=1, m=6) | 16  (f=9, m=7) | 36  (f=13, m=23) |
| *BMI (kg/m²)* | 22.95  (SD 2.88) | 24.13  (SD 1.48) | 23.67  (SD 2.22) | 23.48  (SD 2.37) |
| *Age* | 47.41  (SD 7.93) | 49.14  (SD 8.13) | 50.13  (SD 8.58) | 50.2  (SD 8.6) |
| *Weekly running distance (km)* | 85.41  (SD 15.88) | 78.57  (SD 37.27) | 56.80  (SD 22.98) | 71.38  (SD 27.20) |
| *Physical activity levels (IPAQ)*  *Low*  *Moderate*  *High*  *Sitting/day (min.)* | 0  1  9  333.75  (SD 169.00) | 0  2  3  363.42  (SD 273.68) | 0  3  7  345.50  (SD 196.93) | 0  6  19  345.03  (SD 199.43) |
| *Marathons*  *Participated*  *Finished* | 64.25  (SD 60.29)  Median = 35  Min = 0  Max = 178  64.00  (SD 60.29)  Median = 35  Min = 0  Max = 178 | 53.14  (SD 37.52)  Median = 42  Min = 15  Max = 110  51.42  (SD 39.76)  Median = 42  Min = 3  Max = 110 | 53.29  (SD 55.81)  Median = 37  Min = 2 Max = 178  52.71  (SD 55.92)  Median = 40  Min = 2 Max = 178 | 57.24  (SD 52.99)  Median = 35.5  Min = 0  Max = 178  56.55  (SD 53.38)  Median = 35  Min = 0  Max =178 |
| *Ultramarathons*  *Participated*  *Finished* | 56.58  (SD 56.98)  Median = 34  Min = 6  Max = 190  55.08  (SD 55.13)  Median = 33  Min = 5  Max = 180 | 37.57  (SD 30.61)  Median = 18  Min = 12  Max = 87  36.43  (SD 30.54)  Median = 18  Min = 10  Max = 87 | 32.93  (SD 42.23)  Median = 16  Min = 2  Max = 136  31.93  (SD 41.41)  Median = 15  Min = 2  Max = 132 | 42.52  (SD 46.22)  Median = 21  Min = 2  Max = 190  41.30  (SD 45.07)  Median = 20  Min = 2  Max = 180 |

Summary of sociodemographic and training characteristics of runners participating in the TTdR 2024, grouped by race distance (230 km, 160 km, 100 km). Values are means with standard deviations (*SD*). *BMI = Body Mass Index; IPAQ = International Physical Activity Questionnaire.* Data were collected on-site during the TTdR 2024 event. n = 36. IPAQ: n = 25; NA = 11.

**Table S4. Runner’s High, Pain, Anxiety, and Exercise Dependence in Ultramarathon Runners at the TTdR 2024 (n = 36)**

|  | TTdR  230 km | TTdR  160 km | TTdR  100 km | Total values |
| --- | --- | --- | --- | --- |
| *Did you ever experience a runner’s high before?*  *Yes*  *No*  *I do not know*  *NA* | 10  0  3  0 | 4  0  3  0 | 10  1  3  2 | 24  1  9  2 |
| *How often do you experience a runner’s high under normal running conditions?*  *Always*  *Frequently*  *Rarely*  *Very rarely*  *Never*  *NA* | 0  4  4  1  2  2 | 0  1  3  3  0  0 | 0  4  7  1  3  1 | 0  9  14  5  5  3 |
| *Did you experience a runner's high?*  *Yes*  *No*  *I do not know* | 6  6  1 | 2  3  2 | 4  10  1 | 12  19  5 |
| *Did you experience pain during running?*  *Yes*  *No*  *I do not know*  *NA* | 12  1  0  0 | 4  2  1  0 | 13  2  0  1 | 29  5  1  1 |
| *Did you take any pain medication during the competition?*  *Yes*  *No* | 5  8 | 2  5 | 1  15 | 8  28 |
| *Did you experience anxiety during running?*  *Yes*  *No*  *I do not know*  *NA* | 4  9  0  0 | 0  6  1  0 | 1  14  0  1 | 5  29  1  1 |
| *EDS-R*  *At-risk for exercise dependence*  *Non-dependent but symptomatic*  *Non-dependent*  *NA* | 0  12  1  0 | 0  5  0  2 | 0  14  1  1 | 0  31  2  3 |

Self-reported experiences of runner’s high, pain, anxiety, pain medication use, and exercise dependence (EDS-R) among runners participating in the TTdR 2024, stratified by race distance (230 km, 160 km, 100 km). Values represent absolute frequencies. TTdR = TorTour de Ruhr®; EDS-R = Exercise Dependence Scale-Revised; NA = no answer. Data were collected on-site during the TTdR 2024 event.

| **Table S5:** Inferential statistics for the LME analysis examining the effect of condition and time point on AEA in the marathon study | | | | | | |
| --- | --- | --- | --- | --- | --- | --- |
| Fixed Effects | | | | | | |
|  |  | *Est.*/β | *SE* | *df* | *t* | *p* |
| (Intercept) | | 1.62 | 0.13 | 24.45 | 12.94 | < .001 |
| condition | | -0.20 | 0.19 | 32.53 | -1.10 | .281 |
| timepoint 2 | | 0.91 | 0.08 | 137.44 | 11.59 | < .001 |
| timepoint 3 | | 1.18 | 0.08 | 137.10 | 15.56 | < .001 |
| timepoint 4 | | 1.30 | 0.08 | 137.37 | 16.71 | < .001 |
| timepoint 5 | | 1.04 | 0.08 | 137.10 | 13.72 | < .001 |
| condition × timepoint 2 | | 0.56 | 0.16 | 137.42 | 3.57 | < .001 |
| condition × timepoint 3 | | 0.69 | 0.15 | 137.10 | 4.53 | < .001 |
| condition × timepoint 4 | | 1.00 | 0.16 | 137.36 | 6.41 | < .001 |
| condition × timepoint 5 | | 0.88 | 0.15 | 137.10 | 5.79 | < .001 |
| Random Effects | | | | | | |
|  |  | *Variance* | *SD* | *Corr* | | |
| subject (intercept) | | 0.24 | 0.49 |  |  |  |
| subject (condition) | | 0.44 | 0.66 | 0.27 |  |  |
| residual | | 0.11 | 0.33 |  |  |  |
| Model Fit | | | | | | |
| *R^2^* | marginal | | | conditional | | |
|  | 0.39 | | | 0.86 | | |
| post-hoc simple slope analyses | | | | | | |
| level | contrast | *Est.*/β | *SE* | *t* | *df* | *p* |
| T1 | Walking − Marathon | 0.20 | 0.19 | 1.10 | 32.60 | .281 |
| T2 | Walking − Marathon | -0.36 | 0.19 | 1.88 | 35.60 | .069 |
| T3 | Walking − Marathon | -0.48 | 0.19 | 2.61 | 32.60 | .014 |
| T4 | Walking − Marathon | -0.79 | 0.19 | 4.20 | 34.80 | < .001 |
| T5 | Walking − Marathon | -0.67 | 0.19 | 3.63 | 32.60 | .001 |
| Key: p-values for fixed effects calculated using Satterthwaites approximations. Model equation: AEA ~ condition*time point + (1 + condition \| subject) | | | | | | |
| Note. *n*_subjects_ = 19, *n*_observations_ = 183. | | |  |  |  |  |

| **Table S6:** Inferential statistics for the LME analysis examining the effect of condition and time point on 2-AG in the marathon study | | | | | | |
| --- | --- | --- | --- | --- | --- | --- |
| Fixed Effects | | | | | | |
|  |  | *Est.*/β | *SE* | *df* | *t* | *p* |
| (Intercept) | | 0.44 | 0.07 | 26.26 | 6.44 | < .001 |
| condition | | -0.45 | 0.08 | 74.71 | -5.89 | < .001 |
| timepoint 2 | | 0.03 | 0.05 | 134.91 | 0.55 | .580 |
| timepoint 3 | | 0.06 | 0.05 | 133.63 | 1.37 | .170 |
| timepoint 4 | | 0.22 | 0.05 | 134.27 | 4.87 | < .001 |
| timepoint 5 | | 0.17 | 0.05 | 133.96 | 3.76 | < .001 |
| condition × timepoint 2 | | -0.20 | 0.09 | 134.77 | -2.19 | .030 |
| condition × timepoint 3 | | 0.08 | 0.09 | 133.63 | 0.88 | .381 |
| condition × timepoint 4 | | 0.68 | 0.09 | 134.27 | 7.43 | < .001 |
| condition × timepoint 5 | | 0.64 | 0.09 | 133.96 | 7.10 | < .001 |
| Random Effects | | | | | | |
|  |  | *Variance* | *SD* | *Corr* | | |
| subject (intercept) | | 0.07 | 0.26 |  |  |  |
| subject (condition) | | 0.03 | 0.18 | 0.14 |  |  |
| residual | | 0.04 | 0.19 |  |  |  |
| Model Fit | | | | | | |
| *R^2^* | marginal | | | conditional | | |
|  | 0.3 | | | 0.8 | | |
| post-hoc simple slope analyses | | | | | | |
| level | contrast | *Est.*/β | *SE* | *t* | *df* | *p* |
| T1 | Walking − Marathon | 0.45 | 0.08 | 5.89 | 76.90 | < .001 |
| T2 | Walking − Marathon | 0.65 | 0.08 | 8.18 | 86.50 | < .001 |
| T3 | Walking − Marathon | 0.37 | 0.07 | 4.91 | 74.70 | < .001 |
| T4 | Walking − Marathon | -0.24 | 0.08 | 3.05 | 81.50 | .003 |
| T5 | Walking − Marathon | -0.20 | 0.08 | 2.58 | 76.90 | .012 |
| Key: p-values for fixed effects calculated using Satterthwaites approximations. Model equation: 2-AG ~ condition*time point + (1 + condition \| subject) | | | | | | |
| Note. *n*_subjects_ = 19, *n*_observations_ = 180. | | |  |  |  |  |

| **Table S7:** Inferential statistics for the LME analysis examining the effect of condition and time point on 1-AG in the marathon study | | | | | | |
| --- | --- | --- | --- | --- | --- | --- |
| Fixed Effects | | | | | | |
|  |  | *Est.*/β | *SE* | *df* | *t* | *p* |
| (Intercept) | | 1.09 | 0.13 | 24.97 | 8.42 | < .001 |
| condition | | -0.50 | 0.15 | 52.40 | -3.24 | .002 |
| timepoint 2 | | -0.02 | 0.08 | 138.45 | -0.29 | .773 |
| timepoint 3 | | -0.09 | 0.08 | 138.06 | -1.18 | .241 |
| timepoint 4 | | 0.00 | 0.08 | 138.58 | -0.03 | .974 |
| timepoint 5 | | -0.08 | 0.08 | 138.06 | -0.97 | .333 |
| condition × timepoint 2 | | -0.29 | 0.16 | 138.49 | -1.77 | .078 |
| condition × timepoint 3 | | 0.31 | 0.16 | 138.06 | 1.99 | .049 |
| condition × timepoint 4 | | 0.92 | 0.16 | 138.58 | 5.66 | < .001 |
| condition × timepoint 5 | | 0.77 | 0.16 | 138.06 | 4.84 | < .001 |
| Random Effects | | | | | | |
|  |  | *Variance* | *SD* | *Corr* | | |
| subject (intercept) | | 0.26 | 0.51 |  |  |  |
| subject (condition) | | 0.20 | 0.45 | 0.48 |  |  |
| residual | | 0.12 | 0.34 |  |  |  |
| Model Fit | | | | | | |
| *R^2^* | marginal | | | conditional | | |
|  | 0.12 | | | 0.76 | | |
| post-hoc simple slope analyses | | | | | | |
| level | contrast | *Est.*/β | *SE* | *t* | *df* | *p* |
| T1 | Walking − Marathon | 0.50 | 0.15 | 3.24 | 52.50 | .002 |
| T2 | Walking − Marathon | 0.78 | 0.15 | 5.06 | 54.10 | < .001 |
| T3 | Walking − Marathon | 0.18 | 0.15 | 1.20 | 51.00 | .237 |
| T4 | Walking − Marathon | -0.42 | 0.16 | 2.72 | 55.70 | .009 |
| T5 | Walking − Marathon | -0.27 | 0.15 | 1.79 | 51.00 | .079 |
| Key: p-values for fixed effects calculated using Satterthwaites approximations. Model equation: 1-AG ~ condition*time point + (1 + condition \| subject) | | | | | | |
| Note. *n*_subjects_ = 19, *n*_observations_ = 184. | | |  |  |  |  |

| **Table S8:** Inferential statistics for the LME analysis examining the effect of condition and time point on AA in the marathon study | | | | | | |
| --- | --- | --- | --- | --- | --- | --- |
| Fixed Effects | | | | | | |
|  |  | *Est.*/β | *SE* | *df* | *t* | *p* |
| (Intercept) | | 7.26 | 0.09 | 38.91 | 79.30 | < .001 |
| condition | | -0.13 | 0.14 | 77.48 | -0.92 | .360 |
| timepoint 2 | | 1.20 | 0.09 | 137.85 | 14.05 | < .001 |
| timepoint 3 | | 1.71 | 0.08 | 137.33 | 20.38 | < .001 |
| timepoint 4 | | 1.63 | 0.09 | 138.31 | 18.84 | < .001 |
| timepoint 5 | | 1.09 | 0.09 | 137.64 | 12.91 | < .001 |
| condition × timepoint 2 | | 0.26 | 0.17 | 137.85 | 1.55 | .123 |
| condition × timepoint 3 | | 0.48 | 0.17 | 137.33 | 2.88 | .005 |
| condition × timepoint 4 | | 0.49 | 0.17 | 138.24 | 2.81 | .006 |
| condition × timepoint 5 | | 0.64 | 0.17 | 137.64 | 3.77 | < .001 |
| Random Effects | | | | | | |
|  |  | *Variance* | *SD* | *Corr* | | |
| subject (intercept) | | 0.09 | 0.30 |  |  |  |
| subject (condition) | | 0.11 | 0.33 | -0.39 |  |  |
| residual | | 0.13 | 0.37 |  |  |  |
| Model Fit | | | | | | |
| *R^2^* | marginal | | | conditional | | |
|  | 0.62 | | | 0.8 | | |
| post-hoc simple slope analyses | | | | | | |
| level | contrast | *Est.*/β | *SE* | *t* | *df* | *p* |
| T1 | Walking − Marathon | 0.13 | 0.14 | 0.92 | 77.10 | .360 |
| T2 | Walking − Marathon | -0.14 | 0.14 | 0.94 | 81.80 | .351 |
| T3 | Walking − Marathon | -0.35 | 0.14 | 2.51 | 77.10 | .014 |
| T4 | Walking − Marathon | -0.36 | 0.15 | 2.42 | 86.70 | .018 |
| T5 | Walking − Marathon | -0.51 | 0.14 | 3.57 | 79.40 | < .001 |
| Key: p-values for fixed effects calculated using Satterthwaites approximations. Model equation: AA ~ condition*time point + (1 + condition \| subject) | | | | | | |
| Note. *n*_subjects_ = 19, *n*_observations_ = 183. | | |  |  |  |  |

| **Table S9:** Inferential statistics for the LME analysis examining the effect of condition and time point on PEA in the marathon study | | | | | | |
| --- | --- | --- | --- | --- | --- | --- |
| Fixed Effects | | | | | | |
|  |  | *Est.*/β | *SE* | *df* | *t* | *p* |
| (Intercept) |  | 1.54 | 0.07 | 26.03 | 23.38 | < .001 |
| condition |  | 0.00 | 0.10 | 36.83 | -0.02 | .981 |
| timepoint 2 |  | 0.40 | 0.04 | 135.32 | 8.97 | < .001 |
| timepoint 3 |  | 0.60 | 0.04 | 135.22 | 13.47 | < .001 |
| timepoint 4 |  | 0.56 | 0.05 | 135.47 | 12.48 | < .001 |
| timepoint 5 |  | 0.55 | 0.04 | 135.03 | 12.50 | < .001 |
| condition × timepoint 2 | | 0.13 | 0.09 | 135.30 | 1.47 | .145 |
| condition × timepoint 3 | | 0.07 | 0.09 | 135.22 | 0.84 | .401 |
| condition × timepoint 4 | | 0.15 | 0.09 | 135.44 | 1.70 | .092 |
| condition × timepoint 5 | | 0.05 | 0.09 | 135.03 | 0.59 | .557 |
| Random Effects | | | | | | |
|  |  | *Variance* | *SD* | *Corr* | | |
| subject (intercept) | | 0.06 | 0.25 |  |  |  |
| subject (condition) | | 0.11 | 0.33 | 0.14 |  |  |
| residual |  | 0.04 | 0.19 |  |  |  |
| Model Fit | | | | | | |
| *R^2^* | marginal | | | conditional | | |
|  | 0.3 | | | 0.8 | | |
| post-hoc simple slope analyses | | | | | | |
| level | contrast | *Est.*/β | *SE* | *t* | *df* | *p* |
| — | T1 − T2 | -0.40 | 0.04 | 8.97 | 135.00 | < .001 |
| — | T1 − T3 | -0.60 | 0.04 | 13.47 | 135.00 | < .001 |
| — | T1 − T4 | -0.56 | 0.05 | 12.48 | 135.00 | < .001 |
| — | T1 − T5 | -0.55 | 0.04 | 12.50 | 135.00 | < .001 |
| — | T2 − T3 | -0.20 | 0.05 | 4.31 | 135.00 | < .001 |
| — | T2 − T4 | -0.16 | 0.04 | 3.51 | 136.00 | .003 |
| — | T2 − T5 | -0.14 | 0.04 | 3.21 | 135.00 | .007 |
| — | T3 − T4 | 0.03 | 0.05 | 0.73 | 136.00 | .935 |
| — | T3 − T5 | 0.05 | 0.04 | 1.18 | 135.00 | .718 |
| — | T4 − T5 | 0.02 | 0.05 | 0.42 | 135.00 | .935 |
| Key: p-values for fixed effects calculated using Satterthwaites approximations. Model equation: PEA ~ condition*time point + (1 + condition \| subject) | | | | | | |
| Note. nsubjects = 19, nobservations = 181. | | | |  |  |  |

| **Table S10:** Inferential statistics for the LME analysis examining the effect of condition and time point on AEA in the ultramarathon study | | | | | | |
| --- | --- | --- | --- | --- | --- | --- |
| Fixed Effects | | | | | | |
|  |  | *Est.*/β | *SE* | *df* | *t* | *p* |
| (Intercept) | | 1.85 | 0.11 | 31.93 | 16.63 | < .001 |
| condition 160 | | -0.08 | 0.19 | 31.18 | -0.40 | .689 |
| condition 230 | | 0.06 | 0.16 | 31.35 | 0.36 | .722 |
| timepoint | | 1.73 | 0.19 | 31.72 | 9.30 | < .001 |
| condition 160: timepoint | | 0.01 | 0.32 | 30.99 | 0.02 | .985 |
| condition 230: timepoint | | 0.05 | 0.26 | 31.16 | 0.20 | .847 |
| Random Effects | | | | | | |
|  |  | *Variance* | *SD* |  | | |
| subject (intercept) | | 0.05 | 0.23 |  |  |  |
| residual |  | 0.23 | 0.48 |  |  |  |
| Model Fit | | | | | | |
| *R^2^* | marginal | | | conditional | | |
|  | 0.73 | | | 0.78 | | |
| Key: p-values for fixed effects calculated using Satterthwaites approximations. Model equation: AEA ~ condition*time point + (1 \| subject) | | | | | | |
| Note. *n*_subjects_ = 34, *n*_observations_ = 67. | | | |  |  |  |

| **Table S11:** Inferential statistics for the LME analysis examining the effect of condition and time point on 2-AG in the ultramarathon study | | | | | | |  |
| --- | --- | --- | --- | --- | --- | --- | --- |
|  |  |  |  |  |  |  |  |
| Fixed Effects | | | | | | |  |
|  |  | *Est.*/β | *SE* | *df* | *t* | *p* |  |
| (Intercept) | | 0.38 | 0.06 | 30.00 | 6.70 | < .001 |  |
| condition 160 | | -0.02 | 0.09 | 30.00 | -0.18 | .860 |  |
| condition 230 | | -0.19 | 0.08 | 30.00 | -2.41 | .022 |  |
| timepoint | | 0.57 | 0.08 | 30.00 | 7.05 | < .001 |  |
| condition 160: timepoint | | -0.20 | 0.14 | 30.00 | -1.46 | .156 |  |
| condition 230: timepoint | | -0.21 | 0.11 | 30.00 | -1.85 | .074 |  |
| Random Effects | | | | | | |  |
|  |  | *Variance* | *SD* |  | | |  |
| subject (intercept) | | 0.02 | 0.14 |  |  |  |  |
| residual | | 0.04 | 0.20 |  |  |  |  |
| Model Fit | | | | | | |  |
| *R^2^* | marginal | | | conditional | | |  |
|  | 0.49 | | | 0.66 | | |  |
| post-hoc simple slope analyses | | | | | | |  |
| level | contrast | *Est.*/β | *SE* | *t* | *df* | *p* |  |
| — | 100 km − 160 km | 0.02 | 0.10 | 0.18 | 30.00 | .860 |  |
| — | 100 km − 230 km | 0.19 | 0.08 | 2.41 | 30.00 | .066 |  |
| — | 160 km − 230 km | 0.17 | 0.10 | 1.84 | 30.00 | .151 |  |
| Key: p-values for fixed effects calculated using Satterthwaites approximations. Model equation: 2-AG ~ condition*time point + (1 \| subject) | | | | | | |  |
|  |  |  |  |  |  |  |  |
| Note. *n*_subjects_ = 33, *n*_observations_ = 66. | | |  |  |  |  |  |

| **Table S12:** Inferential statistics for the LME analysis examining the effect of condition and time point on 1-AG in the ultramarathon study | | | | | | |  |
| --- | --- | --- | --- | --- | --- | --- | --- |
|  |  |  |  |  |  |  |  |
|  |  | *Est.*/β | *SE* | *df* | *t* | *p* |  |
| (Intercept) | | 1.15 | 0.11 | 30.00 | 10.20 | < .001 |  |
| condition 160 | | 0.17 | 0.19 | 30.00 | 0.92 | .366 |  |
| condition 230 | | -0.38 | 0.16 | 30.00 | -2.36 | .025 |  |
| timepoint | | 0.02 | 0.17 | 30.00 | 0.11 | .915 |  |
| condition 160: timepoint | | -0.09 | 0.29 | 30.00 | -0.30 | .764 |  |
| condition 230: timepoint | | -0.13 | 0.25 | 30.00 | -0.52 | .609 |  |
| Random Effects | | | | | | |  |
|  |  | *Variance* | *SD* |  | | |  |
| subject (intercept) | | 0.07 | 0.26 |  |  |  |  |
| residual | | 0.20 | 0.44 |  |  |  |  |
| Model Fit | | | | | | |  |
| *R^2^* | marginal | | | conditional | | |  |
|  | 0.17 | | | 0.37 | | |  |
| post-hoc simple slope analyses | | | | | | |  |
| level | contrast | *Est.*/β | *SE* | *t* | *df* | *p* |  |
| — | 100 km − 160 km | -0.17 | 0.19 | 0.92 | 30.00 | .366 |  |
| — | 100 km − 230 km | 0.38 | 0.16 | 2.36 | 30.00 | .050 |  |
| — | 160 km − 230 km | 0.55 | 0.19 | 2.90 | 30.00 | .021 |  |
| Key: p-values for fixed effects calculated using Satterthwaites approximations. Model equation: 1-AG ~ condition*time point + (1 \| subject) | | | | | | |  |
|  |  |  |  |  |  |  |  |
| Note. *n*_subjects_ = 33, *n*_observations_ = 66. | | |  |  |  |  |  |

| **Table S13:** Inferential statistics for the LME analysis examining the effect of condition and time point on AA in the ultramarathon study | | | | | | |  |
| --- | --- | --- | --- | --- | --- | --- | --- |
|  |  |  |  |  |  |  |  |
|  |  | *Est.*/β | *SE* | *df* | *t* | *p* |  |
| (Intercept) |  | 7.89 | 0.11 | 31.00 | 68.98 | < .001 |  |
| condition 160 | | -0.06 | 0.20 | 31.00 | -0.29 | .776 |  |
| condition 230 | | -0.24 | 0.16 | 31.00 | -1.45 | .157 |  |
| timepoint |  | 1.75 | 0.18 | 31.00 | 9.64 | < .001 |  |
| condition 160: timepoint | | 0.42 | 0.31 | 31.00 | 1.33 | .194 |  |
| condition 230: timepoint | | 0.15 | 0.26 | 31.00 | 0.56 | .582 |  |
| Random Effects | | | | | | |  |
|  |  | *Variance* | *SD* |  | | |  |
| subject (intercept) | | 0.07 | 0.26 |  |  |  |  |
| residual |  | 0.23 | 0.48 |  |  |  |  |
| Model Fit | | | | | | |  |
| *R^2^* | marginal | | | conditional | | |  |
|  | 0.76 | | | 0.81 | | |  |
| Key: p-values for fixed effects calculated using Satterthwaites approximations. Model equation: AA ~ condition*time point + (1 \| subject) | | | | | | |  |
|  |  |  |  |  |  |  |  |
| Note. *n*_subjects_ = 34, *n*_observations_ = 68. | | |  |  |  |  |  |

| **Table S14:** Inferential statistics for the LME analysis examining the effect of condition and time point on PEA in the ultramarathon study | | | | | | |  |
| --- | --- | --- | --- | --- | --- | --- | --- |
|  |  |  |  |  |  |  |  |
|  |  | *Est.*/β | *SE* | *df* | *t* | *p* |  |
| (Intercept) | | 2.03 | 0.08 | 32.18 | 26.65 | < .001 |  |
| condition 160 | | -0.07 | 0.13 | 30.60 | -0.51 | .613 |  |
| condition 230 | | 0.53 | 0.11 | 30.95 | 4.93 | < .001 |  |
| timepoint | | 0.71 | 0.15 | 32.05 | 4.91 | < .001 |  |
| condition 160: timepoint | | -0.57 | 0.24 | 30.49 | -2.32 | .028 |  |
| condition 230: timepoint | | -1.25 | 0.20 | 30.84 | -6.13 | < .001 |  |
| Random Effects | | | | | | |  |
|  |  | *Variance* | *SD* | *Corr* | | |  |
| subject (intercept) | | 0.01 | 0.09 |  |  |  |  |
| residual |  | 0.14 | 0.37 |  |  |  |  |
| Model Fit | | | | | | |  |
| *R^2^* | marginal | | | conditional | | |  |
|  | 0.51 | | | 0.54 | | |  |
| post-hoc simple slope analyses | | | | | | |  |
| level | contrast | *Est.*/β | *SE* | *t* | *df* | *p* |  |
| — | 100 km − 160 km | 0.07 | 0.13 | 0.51 | 30.40 | .613 |  |
| — | 100 km − 230 km | -0.53 | 0.11 | 4.92 | 30.70 | < .001 |  |
| — | 160 km − 230 km | -0.60 | 0.13 | 4.64 | 29.50 | < .001 |  |
| 100 km | Before − After | -0.71 | 0.15 | 4.90 | 31.80 | < .001 |  |
| 160 km | Before − After | -0.15 | 0.20 | 0.75 | 29.40 | .462 |  |
| 230 km | Before − After | 0.54 | 0.14 | 3.75 | 29.40 | < .001 |  |
| Key: p-values for fixed effects calculated using Satterthwaites approximations. Model equation: PEA ~ condition*time point + (1 \| subject) | | | | | | |  |
|  |  |  |  |  |  |  |  |
| Note. *n*_subjects_ = 34, *n*_observations_ = 66. | | |  |  |  |  |  |

| **Table S15:** Inferential statistics for the LME analysis examining the effect of condition and time point on euphoria in the marathon study | | | | | | |
| --- | --- | --- | --- | --- | --- | --- |
| Fixed Effects | | | | | | |
|  |  | *Est.*/β | *SE* | *df* | *t* | *p* |
| (Intercept) | | 44.23 | 5.42 | 23.91 | 8.16 | < .001 |
| condition | | 4.69 | 5.76 | 60.59 | 0.82 | .418 |
| timepoint 2 | | -0.24 | 3.16 | 140.49 | -0.07 | .941 |
| timepoint 3 | | 1.11 | 3.15 | 140.10 | 0.35 | .725 |
| timepoint 4 | | 9.56 | 3.16 | 140.47 | 3.02 | .003 |
| timepoint 5 | | 8.14 | 3.14 | 140.31 | 2.59 | .011 |
| condition × timepoint 2 | | 10.25 | 6.33 | 140.52 | 1.62 | .108 |
| condition × timepoint 3 | | -0.22 | 6.31 | 140.10 | -0.04 | .972 |
| condition × timepoint 4 | | 12.15 | 6.33 | 140.47 | 1.92 | .057 |
| condition × timepoint 5 | | 16.67 | 6.28 | 140.31 | 2.66 | .009 |
| Random Effects | | | | | | |
|  |  | *Variance* | *SD* | *Corr* | | |
| subject (intercept) | | 462.80 | 21.51 |  |  |  |
| subject (condition) | | 249.50 | 15.79 | -0.39 |  |  |
| residual | | 183.80 | 13.56 |  |  |  |
| Model Fit | | | | | | |
| *R^2^* | marginal | | | conditional | | |
|  | 0.09 | | | 0.76 | | |
| post-hoc simple slope analyses | | | | | | |
| level | contrast | *Est.*/β | *SE* | *t* | *df* | *p* |
| T1 | Walking − Marathon | -4.69 | 5.76 | 0.82 | 60.50 | .418 |
| T2 | Walking − Marathon | -14.94 | 5.76 | 2.60 | 60.50 | .012 |
| T3 | Walking − Marathon | -4.47 | 5.76 | 0.78 | 60.50 | .441 |
| T4 | Walking − Marathon | -16.84 | 5.76 | 2.93 | 60.50 | .005 |
| T5 | Walking − Marathon | -21.37 | 5.70 | 3.75 | 58.70 | < .001 |
| Key: p-values for fixed effects calculated using Satterthwaites approximations. Model equation: euphoria ~ condition*time point + (1 + condition \| subject) | | | | | | |
| Note. *n*_subjects_ = 19, *n*_observations_ = 186. | | |  |  |  |  |

| **Table S16:** Inferential statistics for the LME analysis examining the effect of condition and time point on anxiety in the marathon study | | | | | | |
| --- | --- | --- | --- | --- | --- | --- |
| Fixed Effects | | | | | | |
|  |  | *Est.*/β | *SE* | *df* | *t* | *p* |
| (Intercept) | | 6.78 | 1.54 | 156.00 | 4.41 | < .001 |
| condition | | 4.41 | 2.48 | 156.00 | 1.78 | .077 |
| timepoint 2 | | -1.96 | 1.31 | 156.00 | -1.50 | .137 |
| timepoint 3 | | -3.19 | 1.25 | 156.00 | -2.55 | .012 |
| timepoint 4 | | -3.81 | 1.13 | 156.00 | -3.38 | < .001 |
| timepoint 5 | | -3.84 | 1.14 | 156.00 | -3.37 | < .001 |
| condition × timepoint 2 | | -2.46 | 2.63 | 156.00 | -0.94 | .349 |
| condition × timepoint 3 | | -4.50 | 2.51 | 156.00 | -1.80 | .074 |
| condition × timepoint 4 | | -5.83 | 2.25 | 156.00 | -2.59 | .011 |
| condition × timepoint 5 | | -6.23 | 2.28 | 156.00 | -2.73 | .007 |
| Random Effects | | | | | | |
|  |  | *SD* | *Corr* | | | |
| subject (intercept) | | 4.56 |  |  |  |  |
| subject (condition) | | 4.50 | -0.57 |  |  |  |
| residual | | 6.76 |  |  |  |  |
| Model Fit | | | | | | |
| *R^2^* | marginal | | | conditional | | |
|  | 0.05 | | | 0.39 | | |
| post-hoc simple slope analyses | | | | | | |
| level | contrast | *Est.*/β | *SE* | *t* | *df* | *p* |
| T1 | Walking − Marathon | -4.41 | 2.48 | 1.78 | 156.00 | .077 |
| T2 | Walking − Marathon | -1.95 | 1.70 | 1.15 | 156.00 | .253 |
| T3 | Walking − Marathon | 0.09 | 1.51 | 0.06 | 156.00 | .952 |
| T4 | Walking − Marathon | 1.42 | 1.03 | 1.37 | 156.00 | .172 |
| T5 | Walking − Marathon | 1.81 | 1.09 | 1.67 | 156.00 | .097 |
| Key: p-values for fixed effects calculated based on the model’s residual degrees of freedom (naive approximation). A variance function allowed different residual variances per timepoint using nlme::lme. Model equation: anxiety ~ condition*time point + (1 + condition \| subject) | | | | | | |
| Note. *n*_subjects_ = 19, *n*_observations_ = 184. | | |  |  |  |  |

| **Table S17:** Inferential statistics for the LME analysis examining the effect of condition and time point on pain in the marathon study | | | | | | |
| --- | --- | --- | --- | --- | --- | --- |
| Fixed Effects | | | | | | |
|  |  | *Est.*/β | *SE* | *df* | *t* | *p* |
| (Intercept) | | 1.33 | 0.24 | 35.61 | 5.46 | < .001 |
| condition | | 0.18 | 0.35 | 76.91 | 0.52 | .608 |
| timepoint 2 | | 0.03 | 0.21 | 142.22 | 0.16 | .870 |
| timepoint 3 | | 1.03 | 0.21 | 142.22 | 4.95 | < .001 |
| timepoint 4 | | 1.35 | 0.21 | 142.45 | 6.44 | < .001 |
| timepoint 5 | | 0.96 | 0.21 | 142.22 | 4.64 | < .001 |
| condition × timepoint 2 | | 0.06 | 0.42 | 142.22 | 0.14 | .892 |
| condition × timepoint 3 | | 0.90 | 0.42 | 142.22 | 2.17 | .032 |
| condition × timepoint 4 | | 0.87 | 0.42 | 142.45 | 2.07 | .041 |
| condition × timepoint 5 | | 1.08 | 0.42 | 142.22 | 2.59 | .011 |
| Random Effects | | | | | | |
|  |  | *Variance* | *SD* | *Corr* | | |
| subject (intercept) | | 0.7058 | 0.8401 |  |  |  |
| subject (condition) | | 0.7232 | 0.8504 | -0.15 |  |  |
| residual | | 0.8075 | 0.8986 |  |  |  |
| Model Fit | | | | | | |
| *R^2^* | marginal | | | conditional | | |
|  | 0.23 | | | 0.63 | | |
| post-hoc simple slope analyses | | | | | | |
| level | contrast | β | SE | t | df | p |
| T1 | Walking − Marathon | -0.18 | 0.36 | 0.52 | 77.10 | .608 |
| T2 | Walking − Marathon | -0.24 | 0.35 | 0.68 | 74.90 | .497 |
| T3 | Walking − Marathon | -1.09 | 0.35 | 3.09 | 74.90 | .003 |
| T4 | Walking − Marathon | -1.05 | 0.36 | 2.96 | 77.10 | .004 |
| T5 | Walking − Marathon | -1.26 | 0.35 | 3.59 | 74.90 | < .001 |
| Key: p-values for fixed effects calculated using Satterthwaites approximations. Model equation: pain ~ condition*time point + (1 + condition \| subject) | | | | | | |
| Note. *n*_subjects_ = 19, *n*_observations_ = 188. | | |  |  |  |  |

| **Table S18:** Inferential statistics for the LME analysis examining the effect of condition and time point on euphoria in the ultramarathon study | | | | | | |  |
| --- | --- | --- | --- | --- | --- | --- | --- |
|  |  |  |  |  |  |  |  |
|  |  | *Est.*/β | *SE* | *df* | *t* | *p* |  |
| (Intercept) |  | 74.99 | 4.12 | 35.26 | 18.21 | < .001 |  |
| condition 160 | | -1.66 | 7.25 | 30.96 | -0.23 | .820 |  |
| condition 230 | | -12.30 | 5.78 | 32.09 | -2.13 | .041 |  |
| timepoint |  | 13.17 | 8.00 | 35.14 | 1.65 | .109 |  |
| condition 160: timepoint | | -31.50 | 14.00 | 30.81 | -2.25 | .032 |  |
| condition 230: timepoint | | -13.17 | 11.18 | 31.96 | -1.18 | .248 |  |
| Random Effects | | | | | | |  |
|  |  | *Variance* | *SD* |  | | |  |
| subject (intercept) | | 15.18 | 3.90 |  |  |  |  |
| residual |  | 396.29 | 19.91 |  |  |  |  |
| Model Fit | | | | | | |  |
| *R^2^* | marginal | | | conditional | | |  |
|  | 0.14 | | | 0.18 | | |  |
| Key: p-values for fixed effects calculated using Satterthwaites approximations. Model equation: euphoria ~ condition*time point + (1 \| subject) | | | | | | |  |
|  |  |  |  |  |  |  |  |
| Note. *n*_subjects_ = 34, *n*_observations_ = 63. | | |  |  |  |  |  |

| **Table S19:** Inferential statistics for the LME analysis examining the effect of condition and time point on anxiety in the ultramarathon study | | | | | | |  |
| --- | --- | --- | --- | --- | --- | --- | --- |
|  |  |  |  |  |  |  |  |
|  |  | *Est.*/β | *SE* | *df* | *t* | *p* |  |
| (Intercept) | | 15.98 | 5.07 | 32.00 | 3.15 | .004 |  |
| condition 160 | | 16.52 | 9.26 | 32.00 | 1.79 | .084 |  |
| condition 230 | | 0.95 | 7.31 | 32.00 | 0.13 | .898 |  |
| timepoint | | -30.88 | 8.20 | 27.00 | -3.76 | < .001 |  |
| condition 160: timepoint | | -24.12 | 14.47 | 27.00 | -1.67 | .107 |  |
| condition 230: timepoint | | 16.26 | 11.53 | 27.00 | 1.41 | .170 |  |
| Random Effects | | | | | | |  |
|  |  |  | *SD* |  | | |  |
| subject (intercept) | |  | 12.11 |  |  |  |  |
| residual |  |  | 29.20 |  |  |  |  |
| Model Fit | | | | | | |  |
| *R^2^* | marginal | | | conditional | | |  |
|  | 0.23 | | | 0.35 | | |  |
| post-hoc simple slope analyses | | | | | | |  |
| level | contrast | *Est.*/β | *SE* | *t* | *df* | *p* |  |
| 100 km | Before − After | 30.90 | 8.20 | 3.76 | 27.00 | < .001 |  |
| 160 km | Before − After | 55.00 | 11.90 | 4.61 | 27.00 | < .001 |  |
| 230 km | Before − After | 14.60 | 8.10 | 1.81 | 27.00 | .082 |  |
| Key: p-values for fixed effects calculated based on the model’s residual degrees of freedom (naive approximation). Model equation: anxiety ~ condition*time point + (1 \| subject) | | | | | | |  |
| Note. *n*_subjects_ = 35, *n*_observations_ = 65. | | |  |  |  |  |  |

| **Table S20:** Inferential statistics for the LME analysis examining the effect of condition and time point on pain in the ultramarathon study | | | | | | |  |
| --- | --- | --- | --- | --- | --- | --- | --- |
|  |  |  |  |  |  |  |  |
|  |  | *Est.*/β | *SE* | *df* | *t* | *p* |  |
| (Intercept) |  | 33.95 | 4.62 | 34.83 | 7.34 | < .001 |  |
| condition 160 | | -2.88 | 8.01 | 30.86 | -0.36 | .722 |  |
| condition 230 | | 2.59 | 6.66 | 31.70 | 0.39 | .700 |  |
| timepoint |  | 46.45 | 7.76 | 34.05 | 5.98 | < .001 |  |
| condition 160: timepoint | | -1.45 | 13.11 | 29.99 | -0.11 | .912 |  |
| condition 230: timepoint | | 12.01 | 10.97 | 30.89 | 1.10 | .282 |  |
| Random Effects | | | | | | |  |
|  |  | *Variance* | *SD* |  | | |  |
| subject (intercept) | | 104.30 | 10.21 |  |  |  |  |
| residual |  | 390.30 | 19.76 |  |  |  |  |
| Model Fit | | | | | | |  |
| *R^2^* | marginal | | | conditional | | |  |
|  | 0.58 | | | 0.66 | | |  |
| Key: p-values for fixed effects calculated using Satterthwaites approximations. Model equation: pain ~ condition*time point + (1 \| subject) | | | | | | |  |
| Note. *n*_subjects_ = 36, *n*_observations_ = 67. | | |  |  |  |  |  |

| **Table S21**: Inferential statistics for the LME analysis examining the effect of condition, time point, and sex on AA in the marathon study | | | | | | |
| --- | --- | --- | --- | --- | --- | --- |
| Fixed Effects | | | | | | |
|  |  | *Est.*/β | *SE* | *df* | *t* | *p* |
| (Intercept) |  | 7.05 | 0.28 | 36.09 | 25.31 | < 0.001 |
| condition |  | -0.05 | 0.43 | 70.37 | -0.11 | .916 |
| timepoint 2 |  | 0.78 | 0.25 | 132.47 | 3.05 | .003 |
| timepoint 3 |  | 1.68 | 0.25 | 132.26 | 6.68 | < 0.001 |
| timepoint 4 |  | 1.82 | 0.26 | 133.06 | 6.99 | < 0.001 |
| timepoint 5 |  | 1.77 | 0.25 | 132.26 | 7.03 | < 0.001 |
| sex |  | 0.15 | 0.19 | 36.09 | 0.77 | .446 |
| condition × timepoint 2 | | 0.83 | 0.51 | 132.47 | 1.64 | .104 |
| condition × timepoint 3 | | 0.31 | 0.50 | 132.26 | 0.61 | .544 |
| condition × timepoint 4 | | -0.46 | 0.52 | 132.98 | -0.88 | .379 |
| condition × timepoint 5 | | 0.07 | 0.50 | 132.26 | 0.13 | .894 |
| condition × sex | | -0.06 | 0.30 | 70.37 | -0.21 | .837 |
| timepoint 2 × sex | | 0.28 | 0.17 | 132.37 | 1.61 | .110 |
| timepoint 3 × sex | | 0.02 | 0.17 | 132.26 | 0.10 | .925 |
| timepoint 4 × sex | | -0.16 | 0.18 | 132.69 | -0.89 | .376 |
| timepoint 5 × sex | | -0.48 | 0.17 | 132.26 | -2.75 | .007 |
| condition × timepoint 2 × sex | | -0.36 | 0.35 | 132.37 | -1.04 | .301 |
| condition × timepoint 3 × sex | | 0.13 | 0.35 | 132.26 | 0.37 | .714 |
| condition × timepoint 4 × sex | | 0.73 | 0.35 | 132.65 | 2.07 | .041 |
| condition × timepoint 5 × sex | | 0.37 | 0.35 | 132.26 | 1.08 | .285 |
| Random Effects | | | | | | |
|  |  | *Var* | *SD* | *Corr* | | |
| subject (intercept) | | 0.10 | 0.31 |  |  |  |
| subject (condition) | | 0.12 | 0.35 | -0.42 |  |  |
| residual |  | 0.13 | 0.37 |  |  |  |
| Model Fit | | | | | | |
| *R^2^* | marginal | | | conditional | | |
|  | 0.62 | | | 0.81 | | |
| post-hoc simple slope analyses | | | | | | |
| level | contrast | *Est.*/β | *SE* | *df* | *t* | *p* |
| T1, male | Walking − Marathon | 0.11 | 0.18 | 69.80 | 0.59 | .555 |
| T2, male | Walking − Marathon | -0.36 | 0.18 | 73.20 | -1.99 | .051 |
| T3, male | Walking − Marathon | -0.33 | 0.18 | 69.80 | -1.82 | .073 |
| T4, male | Walking − Marathon | -0.16 | 0.19 | 80.40 | -0.86 | .392 |
| T5, male | Walking − Marathon | -0.33 | 0.18 | 69.80 | -1.86 | .067 |
| T1, female | Walking − Marathon | 0.17 | 0.24 | 69.80 | 0.71 | .478 |
| T2, female | Walking − Marathon | 0.06 | 0.24 | 69.80 | 0.26 | .800 |
| T3, female | Walking − Marathon | -0.40 | 0.24 | 69.80 | -1.68 | .098 |
| T4, female | Walking − Marathon | -0.83 | 0.24 | 69.80 | -3.54 | .001 |
| T5, female | Walking − Marathon | -0.65 | 0.24 | 69.80 | -2.75 | .008 |
| Key: p-values for fixed effects calculated using Satterthwaites approximations. Model equation: AA ~ condition*time point*sex + (1 + condition \| subject) | | | | | | |
| Note. *n*_subjects_ = 19, *n*_observations_ = 186. Model explained significantly more variance than the basic model without sex as an additional factor, χ^2^(10) = 31.77, p < .001. | | | | | | |

**Figure S1: Arachidonic acid concentrations by sex across marathon and ultramarathon conditions**

Shown are circulating arachidonic acid (AA) concentrations (ng/mL) in male and female participants during the marathon (RUN vs. WALK, n = 19) and ultramarathon (100 km, 160 km, 230 km; n = 36) studies. Individual data points represent single participants; error bars indicate means ± standard error of the mean. At baseline, no significant sex differences were observed (all p ≥ 0.449). When sex was included as an additional factor in the linear mixed-effects (LME) models, it did not explain additional variance in AA progression over time in the ultramarathon study, whereas a sex effect emerged for AA in the marathon study.

*AA, arachidonic acid; RUN, running; WALK, walking.*
